# Homogenized finite element analysis of distal tibia sections: Achievements and limitations

**DOI:** 10.1016/j.bonr.2024.101752

**Published:** 2024-03-26

**Authors:** Mathieu Simon, Michael Indermaur, Denis Schenk, Benjamin Voumard, Ivan Zderic, Dominic Mischler, Michael Pretterklieber, Philippe Zysset

**Affiliations:** a ARTORG Centre for Biomedical Engineering Research, University of Bern, Bern, Switzerland; b AO Research Institute Davos, Davos, Switzerland; c Division of macroscopical and clinical Anatomy, Medical University of Graz, Graz, Austria

**Keywords:** Bone, HR-pQCT, Tibia, hFE, Osteoporosis

## Abstract

High-resolution peripheral quantitative computed tomography (HR-pQCT) based micro-finite element (μFE) analysis allows accurate prediction of stiffness and ultimate load of standardised (∼1 cm) distal radius and tibia sections. An alternative homogenized finite element method (hFE) was recently validated to compute the ultimate load of larger (∼2 cm) distal radius sections that include Colles' fracture sites. Since the mechanical integrity of the weight-bearing distal tibia is gaining clinical interest, it has been shown that the same properties can be used to predict the strength of both distal segments of the radius and the tibia. Despite the capacity of hFE to predict structural properties of distal segments of the radius and the tibia, the limitations of such homogenization scheme remain unclear. Therefore, the objective of this study is to build a complete mechanical data set of the compressive behavior of distal segments of the tibia and to compare quantitatively the structural properties with the hFE predictions. As a further aim, it is intended to verify whether hFE is also able to capture the post-yield strain localisation or fracture zones in such a bone section, despite the absence of strain softening in the constitutive model.

Twenty-five fresh-frozen distal parts of tibias of human donors were used in this study. Sections were cut corresponding to an in-house triple-stack protocol HR-pQCT scan, lapped, and scanned using micro computed tomography (μCT). The sections were tested in compression until failure, unloaded and scanned again in μCT. Volumetric bone mineral density (vBMD) and bone mineral content (BMC) were correlated to compression test results. hFE analysis was performed in order to compare computational predictions (stiffness, yield load and plastic deformation field pattern) with the compressive experiment. Namely, strain localization was assessed based on digital volume correlation (DVC) results and qualitatively compared to hFE predictions by comparing mid-slices patterns.

Bone mineral content (BMC) showed a good correlation with stiffness (R^2^ = 0.92) and yield (R^2^ = 0.88). Structural parameters also showed good agreement between the experiment and hFE for both stiffness (R^2^ = 0.96, slope = 1.05 with 95 % CI [0.97, 1.14]) and yield (R^2^ = 0.95, slope = 1.04 [0.94, 1.13]). The qualitative comparison between hFE and DVC strain localization patterns allowed the classification of the samples into 3 categories: bad (15 sections), semi (8), and good agreement (2).

The good correlations between BMC or hFE and experiment for structural parameters were similar to those obtained previously for the distal part of the radius. The failure zones determined by hFE corresponded to registration only in 8 % of the cases. We attribute these discrepancies to local elastic/plastic buckling effects that are not captured by the continuum-based FE approach exempt from strain softening. A way to improve strain localization hFE prediction would be to use longer distal segments with intact cortical shells, as done for the radius. To conclude, the used hFE scheme captures the elastic and yield response of the tibia sections reliably but not the subsequent failure process.

## Graphical abstract

**Figure:**
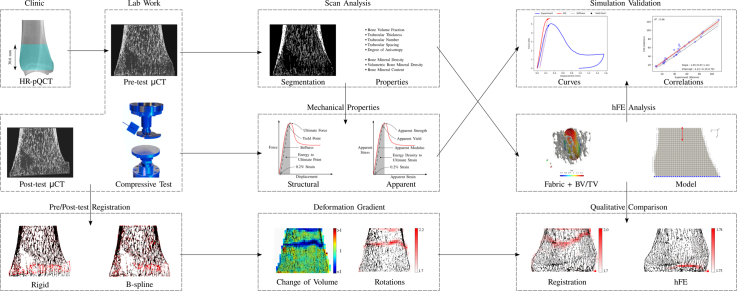

## Abbreviations

HR-pQCT: high resolution peripheral quantitative computed tomography
BMD: bone mass density
ROI: region of interest
BV/TV: bone volume over total volume
hFE: homogenized finite element

## 1 Introduction

Osteoporosis is the most common metabolic bone disease in humans (Sözen et al., 2017). This silent disease is characterized by low bone mass and deteriorated microarchitecture (Sözen et al., 2017) leading to low-energy trauma or even spontaneous fractures. In 2021, the prevalence of osteoporosis in the world was estimated to be 18.3 % (Salari et al., 2021). In Europe, about 32 million people were estimated to have osteoporosis in 2019 and 4.3 million new fragility fractures were recorded (Kanis et al., 2021). These fractures lead to pain, increase morbidity and costs (Johnston and Dagar, 2020). Moreover, fracture treatment requires longer immobilization, accelerating bone loss and further aggravating the severity of the underlying disease (Xidajie, 2009).

The current gold standard for osteoporosis diagnosis is areal bone mineral density (aBMD) evaluation using dual energy x-ray absorptiometry (DXA) (Nuti et al., 2019). This aBMD value is then compared to a young healthy population of the same gender (T-score). According to the WHO, a T-score between −1.0 to −2.5 standard deviation (SD) is defined as osteopenia (low aBMD) and a T-score below −2.5 SD as osteoporosis (WHO, 1994). In fact, the majority of fractures occur in osteopenic rather than in osteoporotic patients which indicates low sensitivity of aBMD in assessing fracture risk (Miller et al., 2002). Indeed, fractures are the consequence of overloading with respect to bone ultimate load, but aBMD does not account for structural integrity, and is, therefore, a limited surrogate of bone strength in fall conditions.

High-resolution peripheral quantitative computed tomography (HR-pQCT) allows for 3D imaging of the bone structure of the peripheral skeleton such as the distal part of the radius and tibia (Boutroy et al., 2005; Gazzotti et al., 2023). Based on HR-pQCT scans, finite element (FE) analysis allows assessing bone strength accurately (Zysset et al., 2013; Rietbergen and Ito, 2015; Engelke et al., 2016). The “gold standard” micro finite element method (μFE) consists of segmenting the HR-pQCT image and converting segmented voxels to hexahedral elements. Such μFE models have shown improving fracture prediction as compared to femoral neck aBMD alone (Samelson et al., 2019).

Nevertheless, μFE models consist of millions of degrees of freedom (DOFs) to solve inducing high computational costs (Rietbergen and Ito, 2015). To reduce the required resources (computational power and time) to estimate bone strength in FE analysis, so-called homogenized finite element (hFE) models were developed (Zysset and Curnier, 1995; Pahr and Zysset, 2009). HR-pQCT together with hFE analysis allows excellent prediction of bone stiffness and strength of the distal segment of the radius (Dünki et al., 2014; Hosseini et al., 2017). Moreover, a more recent study has shown that the same properties can be used to predict stiffness and strength of both the distal segment of the radius and the tibia (Schenk et al., 2022).

Despite the capacity of hFE to predict structural properties of distal segments of the radius and the tibia, the limitations of such homogenization scheme remain unclear. In their work on distal segment of the radius, Varga et al. (2009) raised that hFE could predict more than the global structural response and even capture local strain localization zones. This is of interest since the assessment of local changes could reflect early bone deterioration or, on the opposite, the benefits of a given treatment. One way to investigate hFE capacity to predict strain localization zones is to perform a complete mechanical characterization of the compressive behavior of distal tibia segments and compare it to hFE predictions. This could be done by comparing the global structural responses in a first step and, since multiple field distributions can lead to similar global response, investigating field variables such as strain localization in a second step. To investigate the accuracy of hFE strain localization prediction, a possibility is to use digital volume correlation (DVC) (Bay, 2008). Since the introduction of texture correlation (Bay, 1995), this method has evolved with improved computational and imaging resources, giving rise to the DVC and allowing validation of numerical predictions with experimental measurement (Bay, 2008; Dall'Ara and Tozzi, 2022). Therefore, this study aims to investigate the elastic, yield and post-yield properties of distal tibia sections in compression and make both quantitative and qualitative comparisons with hFE analyses.

## 2 Material and methods

**Fig. 1:**
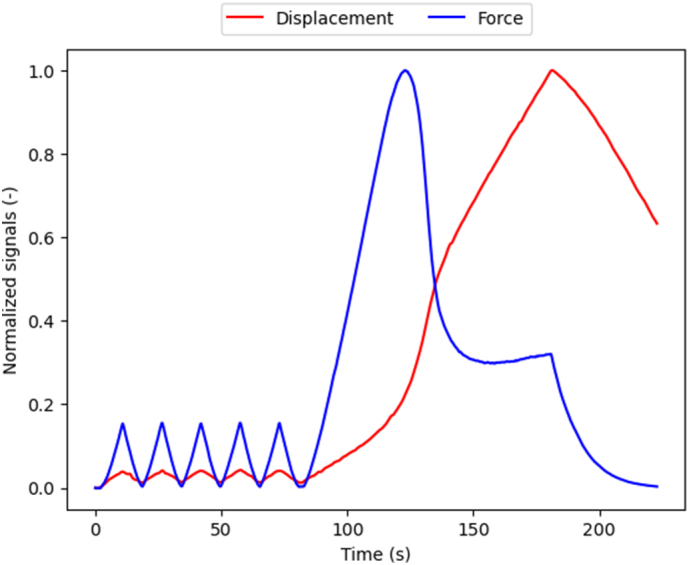
Example of the mechanical testing protocol. The amplitudes of the curves are normalized to improve visualisation.

This study used the tibiae sample presented in Schenk et al. (2022). Briefly, 25 fresh frozen anatomic samples of human tibiae were obtained from the Division of Anatomy of the Medical University of Vienna, Austria, with the authorisation of the local ethics committee. The individuals, who voluntarily donated their bodies for anatomical education and research were 64 to 93 years old at death, with a mean age (± standard deviation) of 82 ± 10 years. Following thawing, the tibiae were scanned using HR-pQCT (XCT II, Scanco Medical, Switzerland) with an in-house protocol (Stuck et al., 2020; Schenk et al., 2020) defined to scan ultradistal sections of about 30.6 mm starting from the cortical bone forming the inferior articular surface of the tibia. The scanned section was then cut out and manually lapped to obtain flat and parallel surfaces. Finally, samples were tested in compression with 5 preconditioning cycles followed by a monotonic loading up to failure, as shown in Fig. 1.

Additionally to Schenk et al. (2022), μCT scans (microCT 100, Scanco Medical AG, Switzerland) were performed before and after mechanical testing in saline solution with an isotropic 24.5 μm voxel size. Scan parameters were for energy: 70 kVp, current: 200 μA and integration time: 300 ms. These scans were then downscaled to a voxel size of 72.5 μm (factor 3) to approximately match the HR-pQCT resolution. These μCT scans allow to first, compare results with clinical HR-pQCT-based hFE, using results of Schenk et al. (2022), and second, to compute the deformation field resulting from the compression experiment.

### 2.1 Image analysis

Pre- and post-test downscaled μCT images were processed using the standard workflow implemented on the scanner's software (IPL Scanco Module 64-bit Version V5.16/FE-v02.02). In a first step, the periosteal contour was defined in a semi-automated way and manually corrected by an operator. Then, the cortical mask, the trabecular mask and the 3 labels segmented image (no bone, trabecular bone, and cortical bone) were generated using a threshold-based algorithm (cortical bone: 450 mgHA/cm^3^, trabecular bone: 320 mgHA/cm^3^) (Whittier et al., 2020) with Gauss filtering (sigma = 0.8, support = 1 voxel). Morphometric analysis was performed using the manufacturer's software on pre and post-test downscaled μCT images as well as on the HR-pQCT scans from Schenk et al. (2022). The resulting parameters, bone volume fraction (BV/TV), trabecular thickness (Tb. Th.), trabecular number (Tb. N.), trabecular spacing (Tb. Sp.), and degree of anisotropy (DA) allows one to compare the samples with the literature (Liu et al., 2010; Manske et al., 2017; Zhou et al., 2016). Additionally, pre-test downscaled μCT gray values scans were converted to bone mineral density (BMD) values using a linear regression performed with a reference phantom scan. Volumetric BMD (vBMD) was computed as mean BMD value of the sample and bone mineral content (BMC) as the sum of BMD values of the segmented image multiplied by the bone volume.

### 2.2 Finite elements analysis

**Fig. 2:**
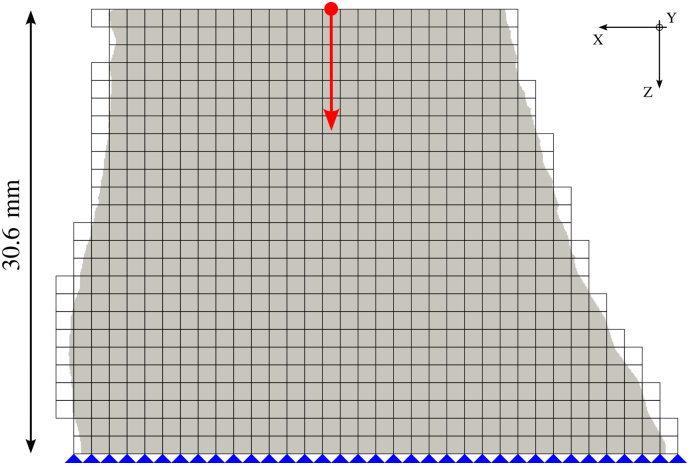
Example of an hFE model. The periosteal mask is in gray, the mesh is in black, and the boundary conditions of the bottom nodes are represented in blue. The virtual reference node is illustrated with the red point and the red arrow is the direction of the imposed displacement.

As field variable prediction highly depends on boundary conditions, downscaled μCT scans performed before the mechanical tests were used for homogenized finite element (hFE) analysis, ensuring simulation of the exact same geometry as the tested sample. Element size was chosen to fit the sample height with a maximum size of 1.27 mm. Therefore, element size varied between 1.20 and 1.25 mm, similar to the size used in the work of Varga et al. (2009). The material homogenization scheme used for the simulations is described by Schenk et al. (2022). Briefly, the fabric and BV/TV of each element were computed within a sphere for which the radius changes according to the bone phase (cortical or trabecular). Then, the corresponding orthotropic stiffness tensor was assigned to the element using the Zysset-Curnier model (Zysset and Curnier, 1995). The scaling factors for stiffness and strength were 0.942 and 0.78, respectively. Moreover, perfect plasticity was used as post-yield behavior (i.e. no softening). Displacements of all bottom nodes (distal surface) were fully constrained. All top nodes (proximal surface) were kinematically coupled to a virtual reference node placed at the top surface along the central vertical axis of the mesh volume. A displacement of 0.3 mm along the vertical direction (∼1% strain) was imposed to the reference node, letting free the 5 other degrees of freedom (Fig. 2). The simulations were performed with the standard solver of Abaqus (Abaqus 6.21-1, Simulia, Dassault Systèmes, Paris, France).

### 2.3 Structural analysis

**Fig. 3:**
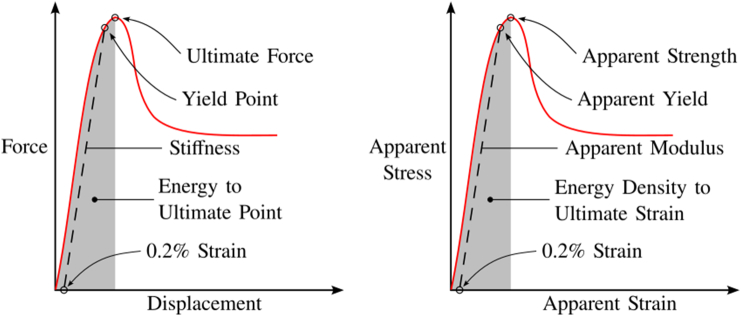
Illustration of the mechanical properties computed from the experiment.

The mechanical test data (force, displacements and rotations) were filtered using a Butterworth filter with a 2.5 Hz cut-off frequency to reduce the noise and synchronized using peak detection. The displacements and rotations applied on the samples were computed as the difference between the top and bottom plates. Stiffness of both experiment and hFE was calculated as the maximum slope of a moving linear regression performed on 1/3 of the data range from the start of the monotonic loading to the maximum load. Then, the yield load was defined with the standard 0.2 % strain yield criterion leading to a displacement of about 0.06 mm. The ultimate load was defined as the absolute maximum force and the energy to the ultimate point as the area under the force-displacement curve up to the ultimate load. The apparent modulus, apparent yield, and apparent strength were computed by dividing sample stiffness, yield, and ultimate load by the samples mean cross sectional area. The apparent strain was computed dividing the displacement by the initial sample height. Finally, the area under the stress-strain curve until the apparent strength was defined as the energy density to ultimate strain (Fig. 3). As experiment and hFE were performed on the exact same geometry, only structural results are presented.

Linear regression analyses were performed and the correlation coefficient was calculated between the mechanical, density-based and hFE results. The relationships between vBMD and sample intensive properties (apparent modulus, apparent yield, and apparent strength) as well as BMC and extensive properties (stiffness, yield, and ultimate load) were assessed. To ensure consistency with clinical HR-pQCT-based hFE analysis, the stiffness results from the tibia sample of Schenk et al. (2022) were correlated to the stiffness resulting from downscaled μCT-based hFE. Then, yield and ultimate load of mechanical tests were correlated. Finally, correlations between hFE and mechanical test were also performed for both stiffness and yield. Linear regression residuals were analysed using box plots and outliers were defined as 1.5 times the interquartile range (IQR) from the first and third residuals quartile (Fox, 2016). The quality of the correlations was assessed using the squared Pearson's correlation coefficient and the standard error of the estimate (Fox, 2016).

### 2.4 DVC for strain localization

Digital volume correlation was performed by registering downscaled μCT scans performed after mechanical test to the ones of the intact samples using SimpleElastix (SimpleITK-SimpleElastix, version 2.0) (Lowekamp et al., 2020) in Python 3.9.7.

As registration can fall into local minima, the procedure should be started with a relatively good initial alignment between the images. Therefore, the registration initial alignment was performed as follow:

1. Alignment of images center of gravity
2. Individual images segmentation using Otsu's method for multi-thresholding (Otsu, 1979) to distinguish 3 classes (background, marrow, and bone), i.e. 2 thresholds. The voxels values below the second threshold were labelled 0 and the others 1, resulting in binary masks
3. Successive rotation the post-test binary mask. After each rotation, Dice coefficient (Dice, 1945), which is usually used to assess similarity between two samples, was computed and the highest value was selected to apply initial rigid rotation to the post-test image

Then, rigid and non-rigid (B-spline) registrations were performed successively on the images. The final interpolation grid for the B-spline registration was set to the same size as the hFE element size to allow comparison. Finally, rigid and b-spline registration quality were quantified using Dice coefficient (Dice, 1945). To compute it, fixed, rigid registered and b-spline registered images were segmented using a common threshold. This threshold was the mean of the second threshold obtained for each image using Otsu's method for multi-thresholding (Otsu, 1979) to again distinguish the same 3 classes.

The deformation gradient (F) was computed from the image registration and extracted from hFE simulation (ABAQUS DFGRD1). Then, volumetric deformation ($\det\left(F\right)$) and the norm of isovolumic deformation ($\left\|\tilde{F}\right\|$) were obtained using the unimodular decomposition of F (Eq. 1). Finally, a qualitative assessment was performed by observing the mid-plane of rigid and B-spline registrations, and the decompositions of F resulting from both the registration and the hFE simulation to assess similarity of strain localization. Results were qualitatively classed into 3 categories: good agreement, partial agreement and bad agreement. A good agreement is defined as an overall correspondence between the strain localization patterns, partial agreement is meant when only part of the strain localization patterns are similar and the sample is said to present a bad agreement when no correspondence can be observed.

(1) $$F=\det{\left(F\right)}^{-1/3}\tilde{F}$$

## 3 Results

### 3.1 Structural response

#### 3.1.1 Mechanical test

Descriptive statistics of the structural values obtained from the mechanical test are presented in Table 1. Apparent properties are available in Appendix B, Table 3. The linear regression analysis showed strong correlation between yield and ultimate load as well as apparent yield and apparent strength (Appendix B, Fig. 10). Relations with yield were similar to those with ultimate force. Therefore, only relations with yield are presented.

**Table 1:** Mechanical structural properties of the tibiae sections tested in compression.

	Stiffness (kN/mm)	Yield force (kN)	Ultimate force (kN)	Ultimate displacement (mm)	Energy to ultimate point (J)
Min–max	9,8–110	1,4–21,1	1,4–21,7	0,17–0,67	0,17–9,52
Mean ± Std	46 ± 26	8,2 ± 5,0	8,5 ± 5,1	0,32 ± 0,12	2,07 ± 2,37

#### 3.1.2 Mechanical test and densitometry

**Fig. 4:**
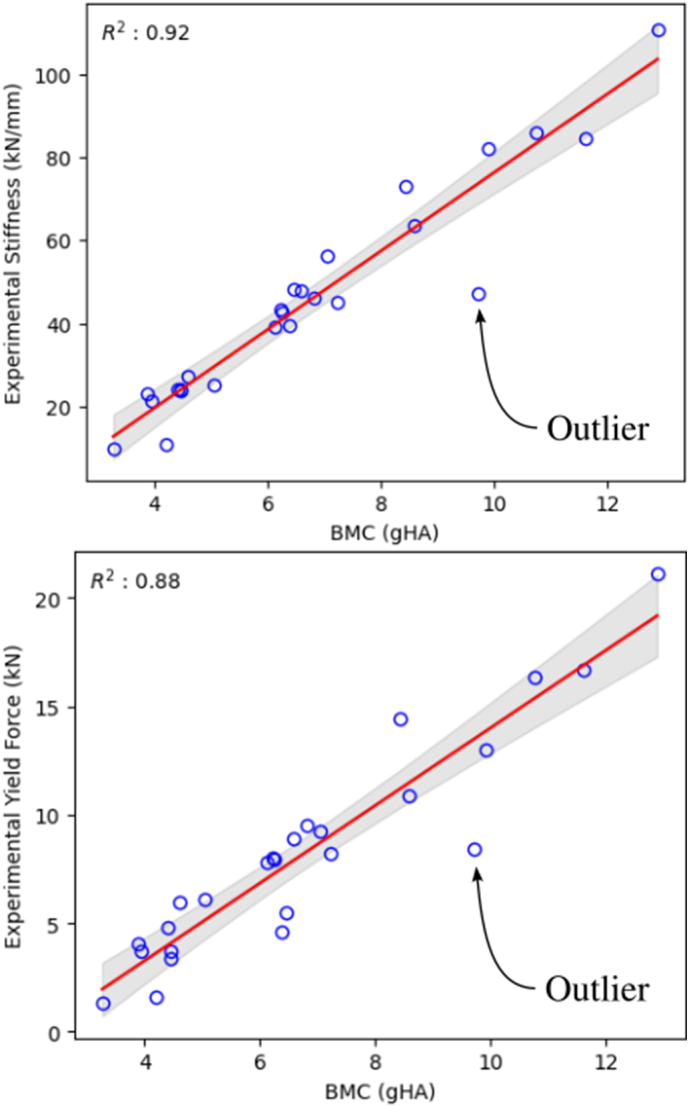
Extensive properties as function of BMC. Top, stiffness as function of BMC and bottom, yield as function of BMC. The red lines show the regression results and the gray area is the 95 % confidence interval.

BMC was strongly correlated with stiffness (R^2^ = 0.92) and yield (R^2^ = 0.88), see Fig. 4. Intensive sample properties (apparent modulus and apparent yield) are shown as function of vBMD in Appendix B, Fig. 11. Analysis of the residuals showed that stiffness, yield, and apparent modulus had common outlier which was also an extreme residual in apparent strength. Analysis of the residuals is also available in Appendix B, Fig. 12.

#### 3.1.3 HR-pQCT hFE and μCT hFE

**Fig. 5:**
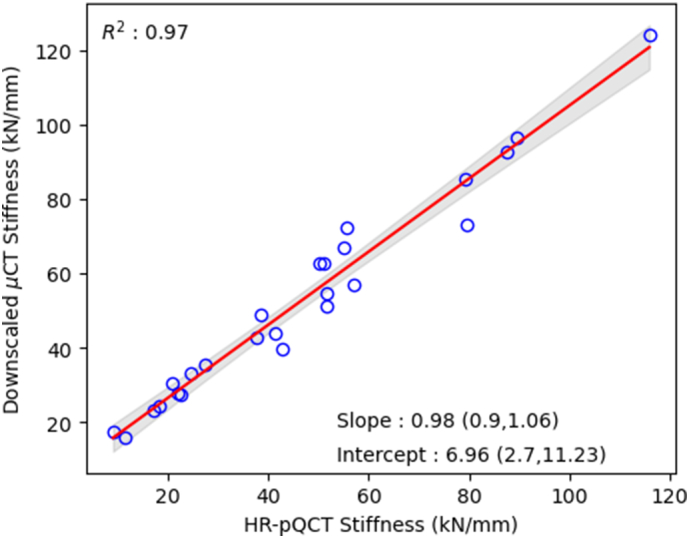
Linear regression between hFE stiffness obtained with downscaled μCT scans and HR-pQCT scans.

Stiffness was strongly correlated between the downscaled μCT based hFE and HR-pQCT-based hFE models (R2 = 0.97) and matched well quantitatively (Fig. 5). Relations regarding apparent modulus are available in Appendix B, Fig. 13.

#### 3.1.4 Mechanical test and hFE

**Fig. 6:**
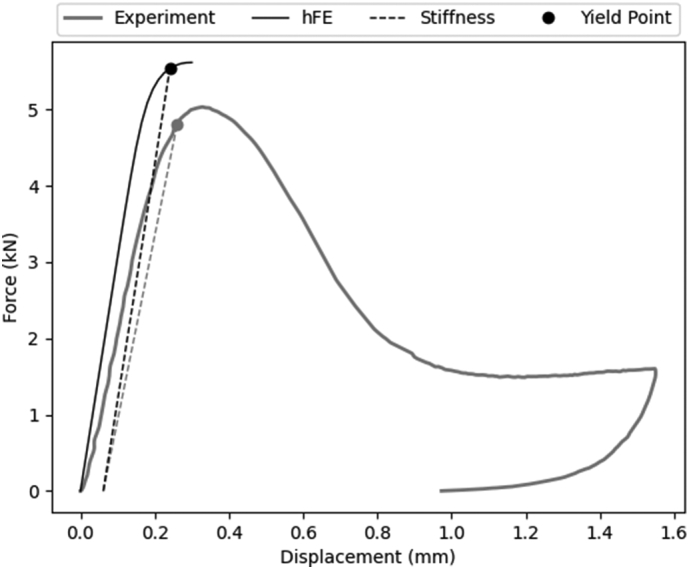
Force-displacement curves of hFE and mechanical test.

Fig. 6 shows the comparison of the force-displacement curves between pre-test downscaled μCT based hFE and mechanical experiment for an exemplary sample. The experimental curves showed an increase of the measured force until reaching a maximum value. Then, the force decreased with increasing displacement and remained relatively constant until another increase. The displacement was then reduced until reaching zero force at the end of the experiment. The hFE curves presented yield but did not show a decrease of the force after reaching the maximum value in all cases. All the force-displacement curves are available in Appendix C.

**Fig. 7:**
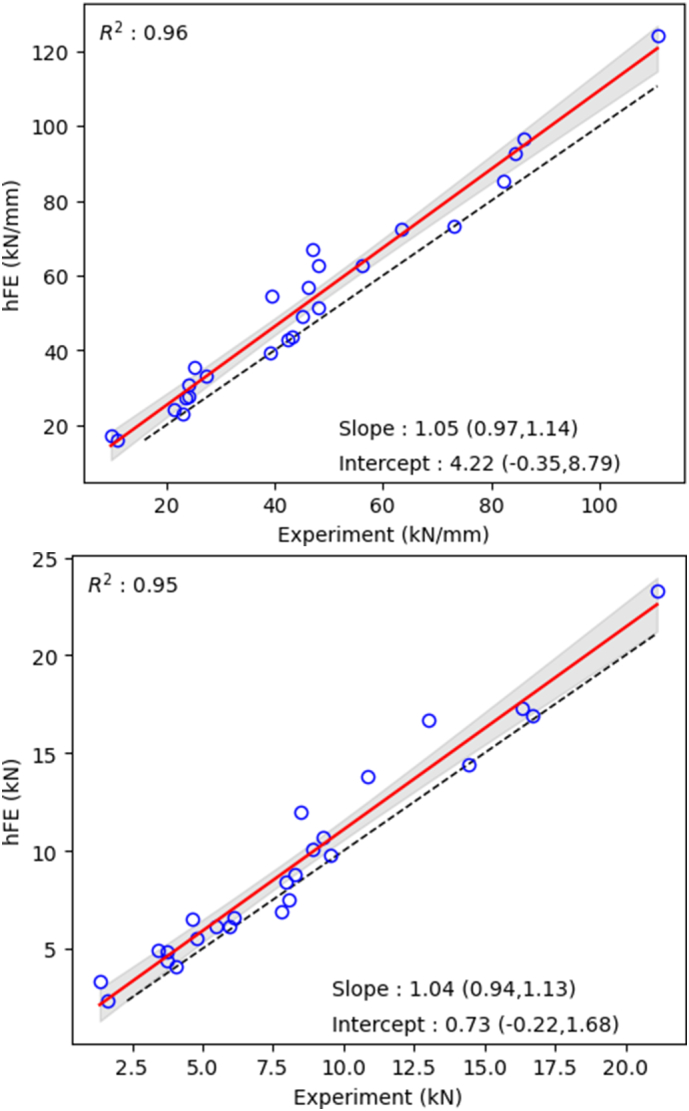
Structural hFE results as function of experimental results. Top, stiffness and bottom, yield. The black dashed line represents the 1:1 line.

Fig. 7 shows the linear regression analyses between the experiment and hFE simulation for the structural response results. Strong correlation were found for both stiffness (R^2^ = 0.96) and yield (R^2^ = 0.95). Moreover, the 95 % CI of the slope and the intercept comprised 1 and 0, respectively, for both stiffness and yield.

### 3.2 Strain localization

**Fig. 8:**
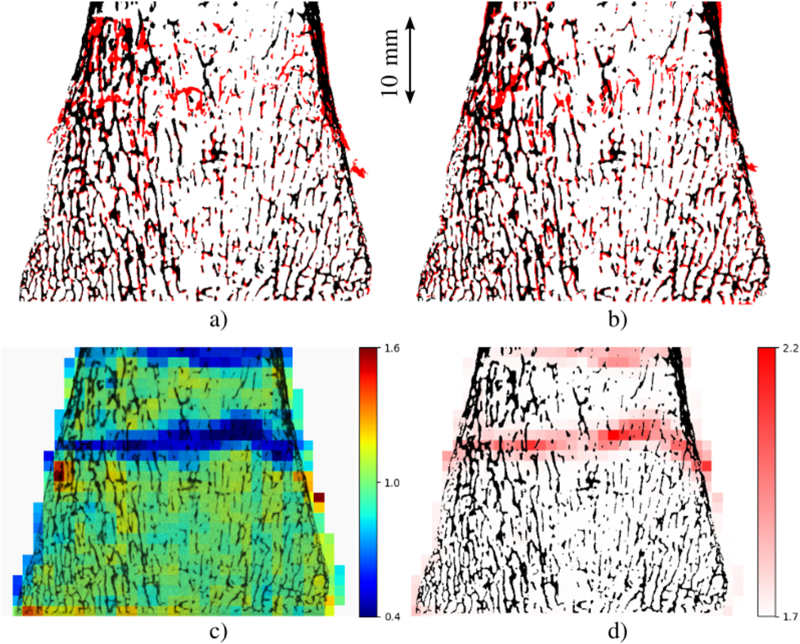
Example of registration results and deformation gradient decomposition. a) Rigid registration b) B-spline registration. White is perfect agreement between μCT pre- and post-registration, red shows pre-test μCT scan and cyan post-test μCT scan. c) Registration $\det\left(F\right)$. d) Registration $\left\|\tilde{F}\right\|$. Results are presented in the original configuration.

Registration between pre- and post-test μCT scans presented an increase of average Dice coefficient from 0.59 (rigid registration) to 0.65 (b-spline registration). The $\det\left(F\right)$ and $\left\|\tilde{F}\right\|$ highlighted the strain localization zones with similar patterns as shown in Fig. 8.

**Fig. 9:**
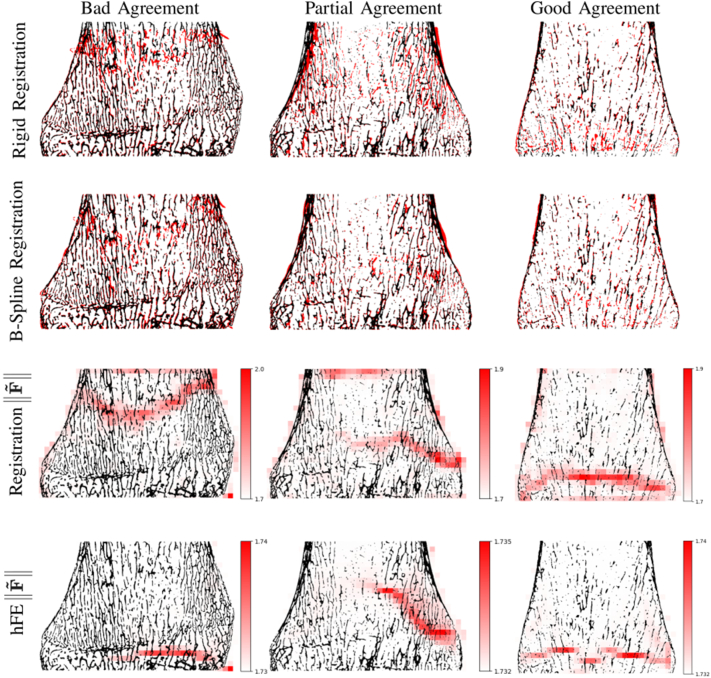
Example of level of agreements between registration and hFE for $\left\|\tilde{F}\right\|$. Results are presented in the original configuration.

Finally, Fig. 9 shows an example for each of the three level of agreement between registration and hFE. Only 8 % (2 samples) showed a good agreement between registration and hFE, 32 % (8 samples) presented a partial agreement and 60 % (15 samples) had a bad agreement between the two methods.

## 4 Discussion and conclusion

This study investigates the compressive behavior of 25 fresh-frozen human distal tibia sections and exposes the achievements as well as the limitations of the hFE scheme beyond the simulation of structural response: the capacity of hFE to predict strain localization, i.e. local field variables. This is performed by looking qualitatively at mid-slice of hFE simulations and pre-/post-test registration. Sixty percent were judged as a bad agreement between hFE and registration, 32 % only presented partial agreement, and a small 8 % presented a good agreement. This contrasts with the results of Varga et al. (2009), performed at the distal part of the radius, where the strain localization zone corresponded fairly well to the hFE predictions. This difference is mainly explained by the different boundary conditions. Indeed, the distal segments of the radii were cut out 4 cm proximal to the distal third and the ultradistal sections were kept intact. This led to ∼4 times longer samples respecting the St-Venant principle regarding the boundary conditions of the proximal side. Regarding the distal side of the sample, the embedding allowed a smooth application of the load and the continuous cortical shell helped to smooth the load distribution within the sample. These conditions, together with the radius presenting a specific weak zone (leading to the Colles' fracture), led to the observed good agreement between strain localization in the experiment and hFE simulation. In the present study, the distal segment of the tibia do not present a specific weaker zone and the boundary conditions impact the results of the local field variables. Moreover, mechanical failure of bone start at the micro-scale with buckling mechanisms which cannot be caught by hFE, averaging properties at the millimeter scale. This highlights a limitation of applying hFE to surgically obtained bone cross sections.

Nevertheless, comparisons between the structural response of the mechanical test and the hFE simulation using downscaled μCT show excellent correlations for both stiffness and yield load (R^2^ = 0.96 and 0.95, respectively). The overestimation of hFE values as compared to experimental results can provide from the voxel approximation of the cortical shell. Indeed, a smoother mesh could improve results in that regards. Such results are similar to other studies performed for the distal segment of the radius (Hosseini et al., 2017; Varga et al., 2011; Arias-Moreno et al., 2019) and distal parts of the radius and tibia (Schenk et al., 2022). Moreover, hFE allows for improvement of stiffness and yield load prediction as compared to densitometric values (e.g. vBMD or BMC), highlighting the importance to use the 3D structure rather than only scalars for bone strength computation. These results confirm, once again, the excellent capacity of the hFE methodology to predict structural response up to the ultimate load.

Correlation between densitometry and mechanical test shows good (R^2^ = 0.82) to excellent (R^2^ = 0.93) correlations for both intensive and extensive properties with vBMD and BMC, respectively. These results are in alignment with similar studies performed at the distal part of the radius (Varga et al., 2011) and to a lower extent slightly better than studies with vertebral bodies (Dall’Ara et al., 2010; Dall’Ara et al., 2012). This confirms the predictive capacity of vBMD and BMC for the intensive and extensive properties of the sample, respectively. Analysis of the residuals of the linear regression highlighted outliers, but no abnormalities were observed on the experimental curves. However, a close examination of rigid registered pre- and post-test μCTs shows that this sample presents an extremely low Ct. Th. on the posterior side. Such a low Ct. Th. explains that the experimentally measured variables are significantly lower than the prediction performed using vBMD or BMC.

The morphometric analysis allows to consider the sample set used in the present study as similar to what exists in the literature, see Appendix A. As some differences exist between HR-pQCT and downscaled μCT scans morphometry, they could lead to differences in hFE simulation which justify a comparison between HR-pQCT hFE results and the ones obtained with downscaled μCT hFE. This comparison presents a high Pearson correlation coefficient (R^2^ = 0.97) for stiffness and a slope of 0.98 with CI containing 1 although the two sections do not correspond at 100 %. This high degree of correlation gives confidence in the similarity of the methods. The linear regression for apparent modulus presents slightly worse results compared to stiffness, highlighting the introduction of additional differences when processing data to obtain apparent properties. This adds confidence in the choice of presenting structural properties rather than apparent properties even if they have the advantage of being are size independent.

The registration procedure used here (with Simple Elastix in Python (Lowekamp et al., 2020)) is able to register highly deformed samples appropriately as observed qualitatively in the results. The fact that the Dice coefficient shows only a moderate increase between rigid and b-spline registration arise from the final interpolation grid. To compare results with hFE, this final interpolation grid is set to the same size as the hFE element size, thus, limiting the registration capacity.

One limitation of the present study is that the simulations were only performed until 1 % strain whereas the compression tests reached higher strains. Nevertheless, the deformation pattern appearing at 1 % strain reflects weaker areas which will undergo strain localization. Moreover, the comparison was performed between only the intact and the final deformation state after unloading. It would be of preference to perform μCT scans while loading and at different strains to improve the understanding of the failure process. Another limitation lies in the fact that only qualitative comparison was performed for strain localization agreement assessment. However, the very low agreement rate releases the importance of quantitative results as the main conclusion will remain the same. Finally, the hFE elements were simple hexahedrons, thus limiting the modelization quality of the cortex shell.

To conclude, this study presents for the first time a complete mechanical data of distal tibiae sections. The results confirm prediction abilities of vBMD and BMC for sample intensive and extensive properties, respectively, as it is shown in hitherto published studies for other anatomical regions. Similarly, hFE stiffness, yield and ultimate load of distal tibia sections are validated by mechanical experiment, highlighting once again the achievements reached with hFE. However, bone failure starts at the micrometer scale which cannot be caught by millimeter scale hFE using a local material model. Finally, this study brings new insight about the abilities and the limitations related to hFE analysis beyond the ultimate point.

## CRediT authorship contribution statement

**Mathieu Simon:** Writing – original draft, Investigation, Formal analysis. **Michael Indermaur:** Writing – review & editing, Supervision, Methodology. **Denis Schenk:** Writing – review & editing, Supervision, Methodology. **Benjamin Voumard:** Writing – review & editing, Supervision, Methodology. **Ivan Zderic:** Writing – review & editing, Resources, Investigation. **Dominic Mischler:** Writing – review & editing, Resources, Investigation. **Michael Pretterklieber:** Writing – review & editing, Resources. **Philippe Zysset:** Writing – review & editing, Supervision, Resources, Project administration, Conceptualization.

## Declaration of competing interest

We wish to confirm that there are no known conflicts of interest associated with this publication and there has been no significant financial support for this work that could have influenced its outcome.

## Data Availability

The data that support the findings of this study are available on request. The data are not publicly available due to privacy/ethical restrictions. The scripts used for the analyses performed in the present study are available on Github: https://github.com/artorg-unibe-ch/FRACTIB.

## Appendix A Supplementary data

Supplementary material Image 1
