# Supplementary material for: Homogenized finite element analysis of distal tibia sections: Achievements and limitations
Source: Bone Rep. 2024 Mar 26;21:101752. doi: 10.1016/j.bonr.2024.101752 (PMC10999809; doi:10.1016/j.bonr.2024.101752)
Supplement: Supplementary file 1 — Supplementary material [file mmc1.pdf]

Study	Device	Voxel Size (μm)	Group Age (Years)	BV/TV (-)	Tb. Th. (mm)	Tb. Sp. (mm)	Tb. N. (1/mm)	DA (-)
Liu et al. [35]	XCT	82	71	0.27 ± 0.05	0.23 ± 0.02	0.73 ± 0.13	1.36 ± 0.22	1.61 ± 0.09
Zhou et al. [36]	XCT	82	72 ± 11	0.23 ± 0.05	0.24 ± 0.02	0.73 ± 0.29	1.44 ± 0.30	1.42 ± 0.08
Present study	XCT II	61	82 ± 10	0.25 ± 0.10	0.22 ± 0.04	0.80 ± 0.40	1.08 ± 0.30	1.74 ± 0.08
	μCT	72.5	82 ± 10	0.17 ± 0.07	0.28 ± 0.05	1.07 ± 0.38	1.04 ± 0.30	1.82 ± 0.10

Table 2
Comparative morphometric values between studies on distal tibiae data sets. Results are presented as mean value ± standard deviation.

A. Morphometry

HR-pQCT morphometric values obtained in the present study can be compared to values obtained in similar studies [35, 36]. Then, μCT values computed on the extracted sample can be compared to the results of the HR-pQCT, as presented in Table 2. First, it should be noted that the present study used a second-generation HR-pQCT scanner whereas the studies used for comparison performed scans with a first-generation HR-pQCT leading to a difference in voxel size. The group age of the present study is different as well of approximately 10 years older. Then, BV/TV, Tb. Th., and Tb. Sp. lay in a similar range as presented in other studies [35, 36]. On the opposite, Tb. N. is lower and DA is higher in the present study. Now comparing HR-pQCT and downscaled μCT of the present study, BV/TV is lower of about 0.12 and Tb. Th. is higher of about 0.06 for downscaled μCT. Tb. Sp. and DA are higher as well but Tb. N. lays in the same range.

The morphometric analysis presents comparable BV/TV values between the present study XCT II scans and other studies XCT scans even if in the present study patients are 10 years older in average. This arises from the different scanning devices leading to different voxel sizes. A previous study has shown that second-generation XCT leads to higher BV/TV as compared to first-generation XCT [37], explaining these similar values. Tb. Th and Tb. Sp. show a good overlap as well between studies as their respective standard deviations ranges overlap. Contrary, the standard deviation of Tb. N of the present study does not overlap with the ones performed with a first-generation HR-pQCT [35, 36] which can be is explained by the different scanning devices used [37]. Finally, DA seems to differ accross studies but is the highest in the present one. This fact is due to the higher resolution in XCT II, as it was shown that higher resolution μCT result in a higher DA as compared to lower resolution XCT [35, 36]. This morphometric analysis allows to consider the sample set used in the present study as similar to what exists in the literature.

Regarding HR-pQCT compared to downscaled μCT, the lower BV/TV and increased Tb. Sp. in downscaled μCT correspond to observations in other studies comparing HR-pQCT scans with μCT scans [35, 36]. The difference in Tb. Th between the HR-pQCT scans and the downscaled μCT scans is of the order of 1 voxel and could arise from the downscaling operation, which coarsens the structure. Tb. N. shows similar values between HR-pQCT scans and downscaled μCT with relatively large standard deviations (about 30% of the mean value) which results in a good overlap. Finally, DA is slightly higher in the downscaled μCT scans. Again, this is due to the lower HR-pQCT resolution as compared to the initial μCT resolution, which results in coarser, bulkier structures.

B. Structural Results

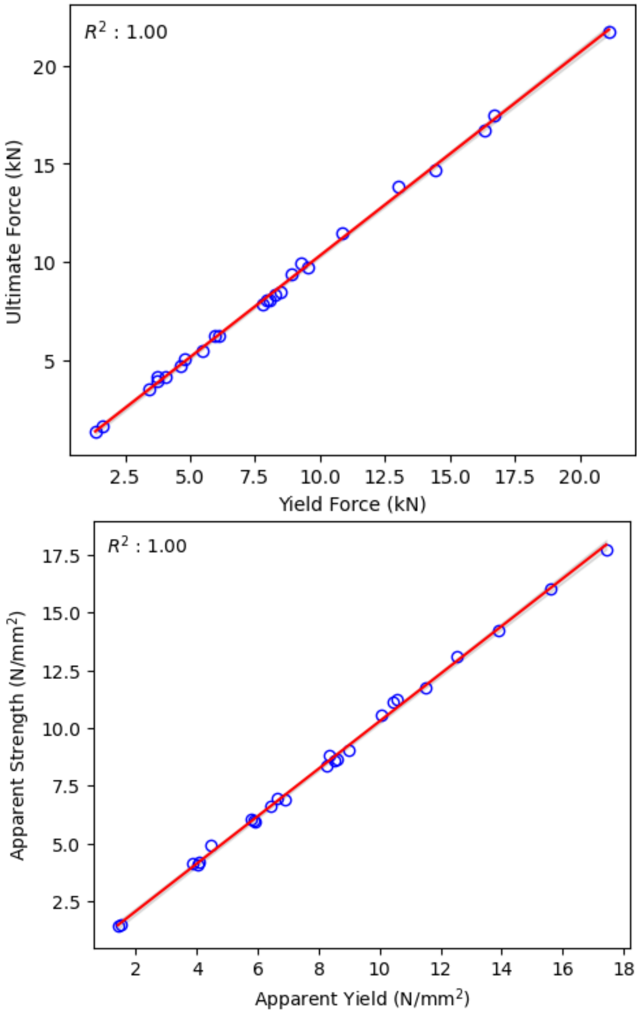

Figure 10: Ultimate force as function of yield force (top) and apparent strength as function of apparent yield (bottom).

	Stiffness (kN/mm)	Yield Force (kN)	Ultimate Force (kN)	Ultimate Displacement (mm)	Energy to Ultimate Point (J)
Min - Max	313 - 2697	1,41 - 17,42	1,41 - 17,73	0,58 - 2,18	5,6 - 243,7
Mean ± Std	45,8 ± 25,6	8,22 ± 4,95	8,49 ± 5,13	0,32 ± 0,12	2,07 ± 2,37

Table 3
Mechanical structural properties of the tibiae sections tested in compression.

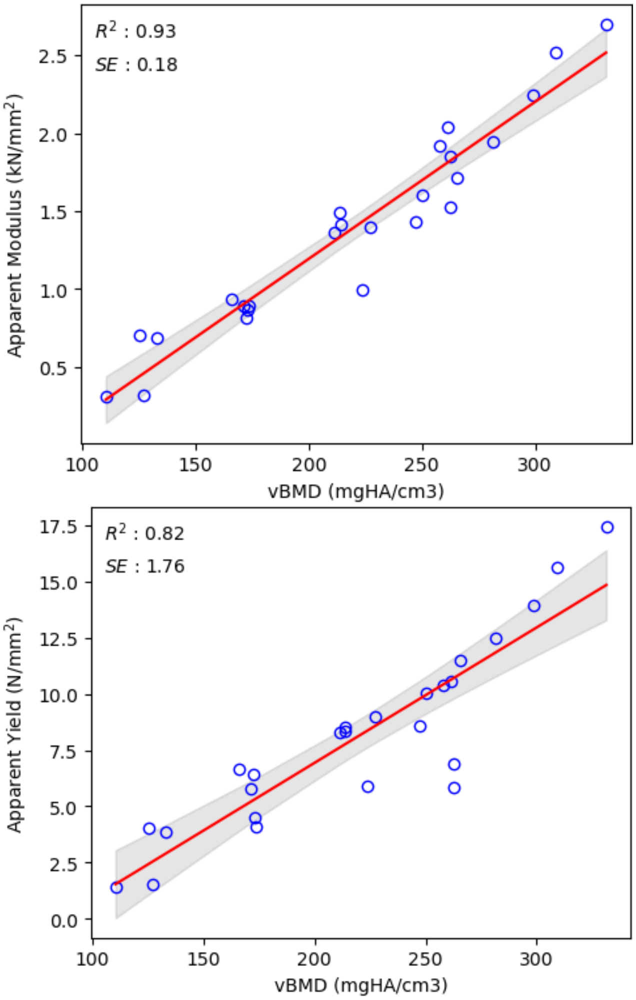

Figure 11: Intensive properties as function of vBMD. Top, apparent modulus as function of vBMD. Bottom, apparent yield as function of vBMD.

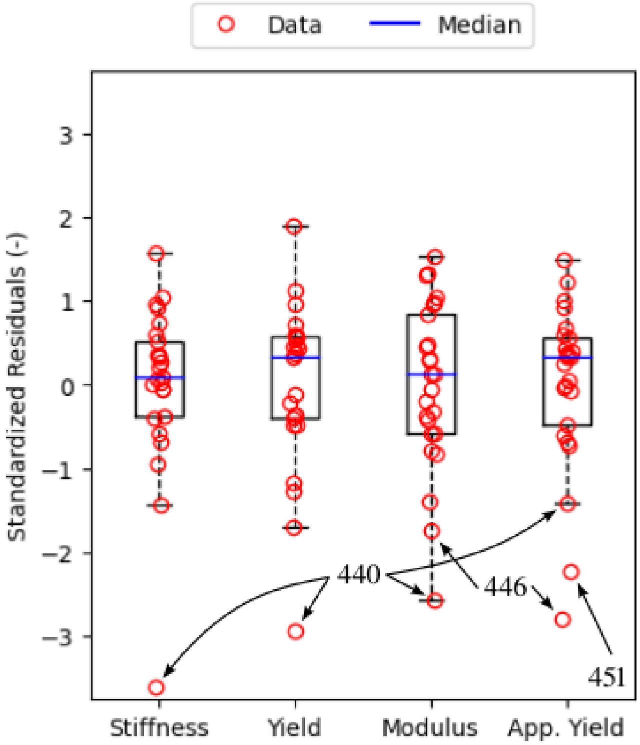

Figure 12: Boxplots of the standardized residuals of linear regressions performed between mechanical properties and densitometry values.

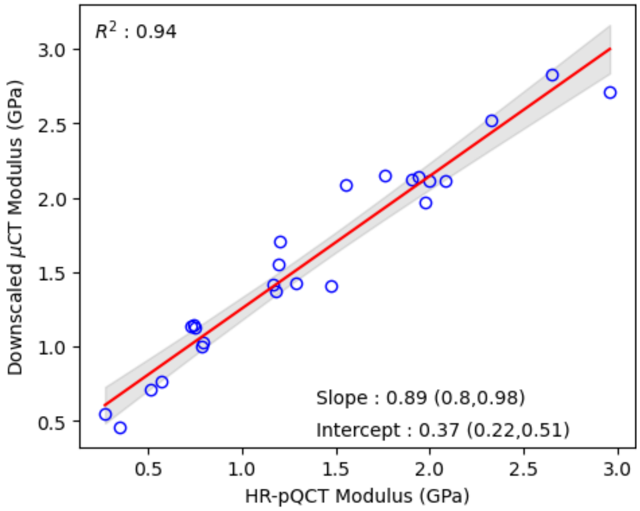

Figure 13: Linear regression between hFE apparent modulus obtained with downscaled µCT scans and HR-pQCT scans.

Figure 12 shows the residuals resulting from the linear regressions performed on extensive (stiffness with BMC and ultimate load with BMC) and intensive (apparent modulus with vBMD and apparent strength with vBMD) variables. For inter-variable comparison, the residuals were standardized with the standard deviation of the respective variable. Stiffness, yield and apparent modulus show all a single outlier, sample number 440 and apparent yield presents two outliers, samples 446 and 451. Strictly speaking, sample 440 is not considered an outlier regarding apparent yield, as it is at the lower limit of the residual quartile.

C. Force-Displacement Curves

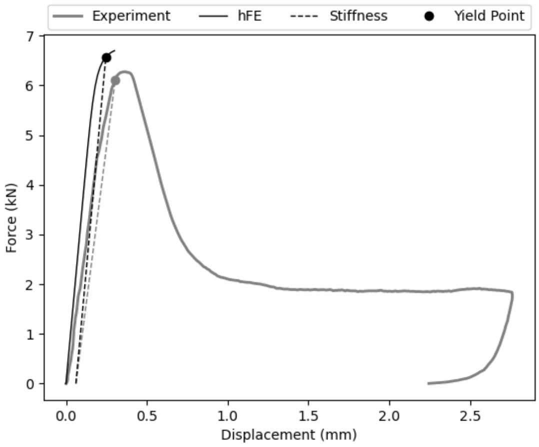

Figure 14: Sample 432

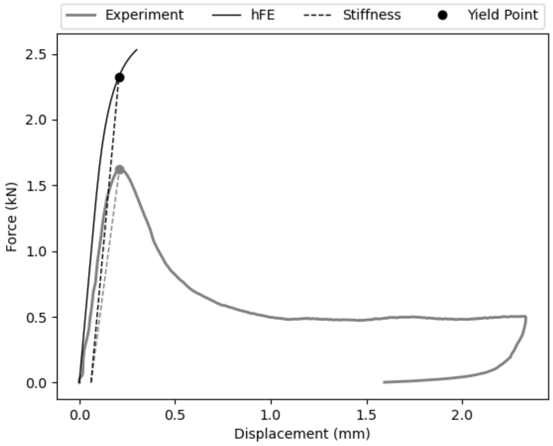

Figure 16: Sample 432

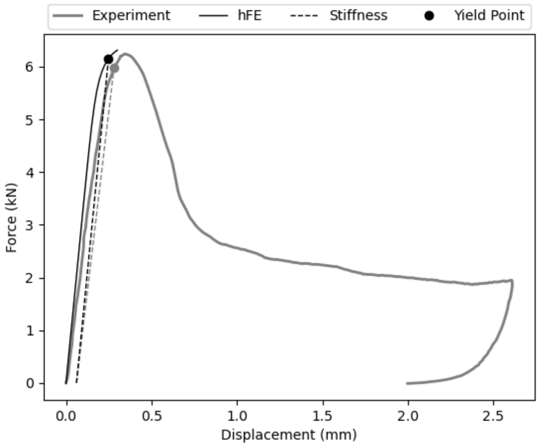

Figure 15: Sample 433

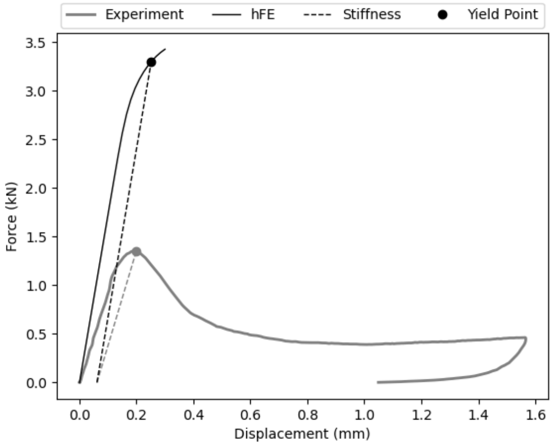

Figure 17: Sample 433

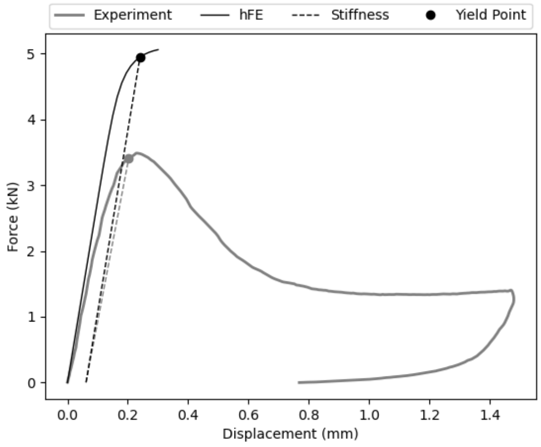

Figure 18: Sample 432

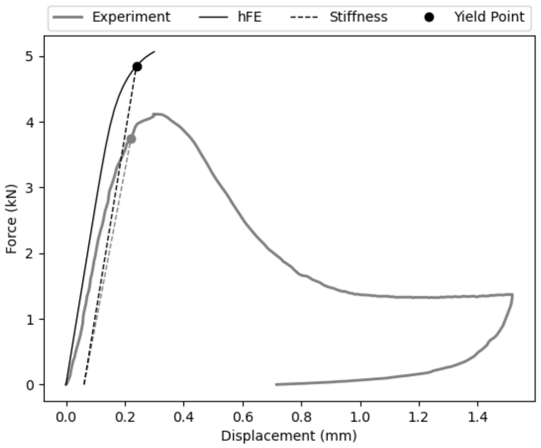

Figure 19: Sample 433

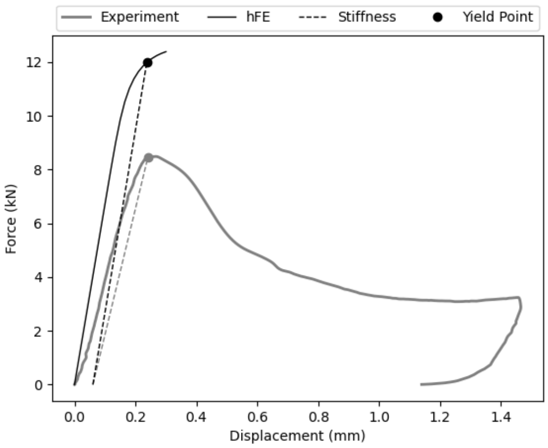

Figure 22: Sample 432

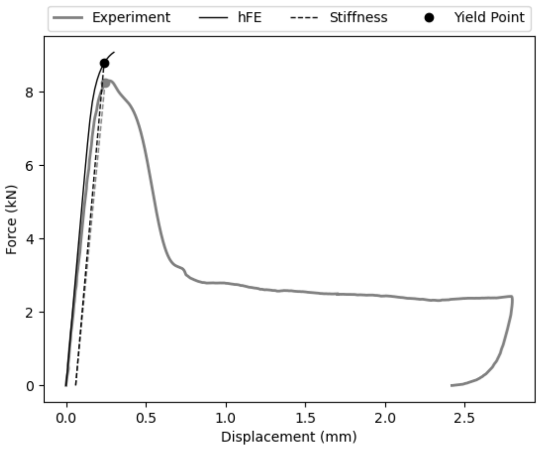

Figure 20: Sample 432

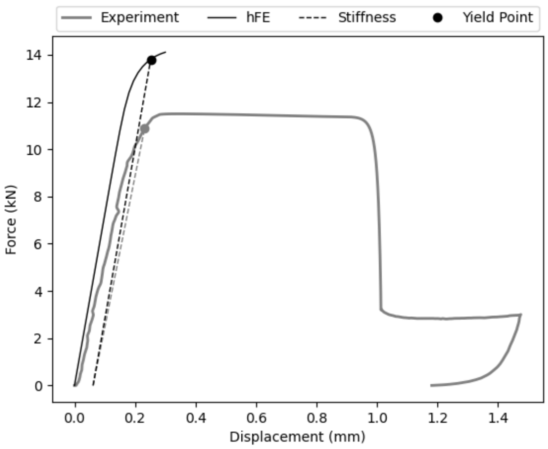

Figure 23: Sample 433

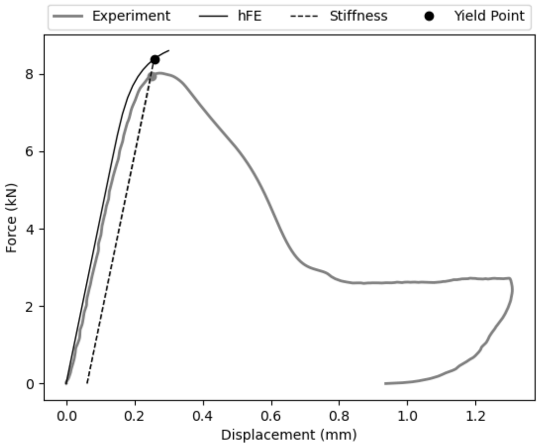

Figure 21: Sample 433

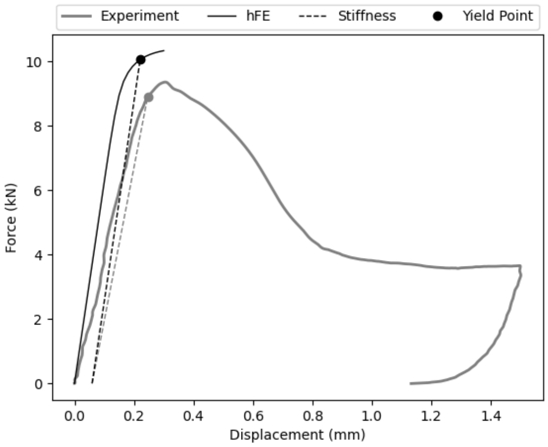

Figure 24: Sample 432

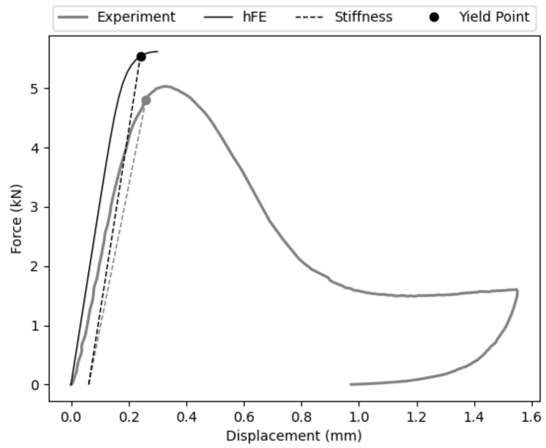

Figure 25: Sample 433

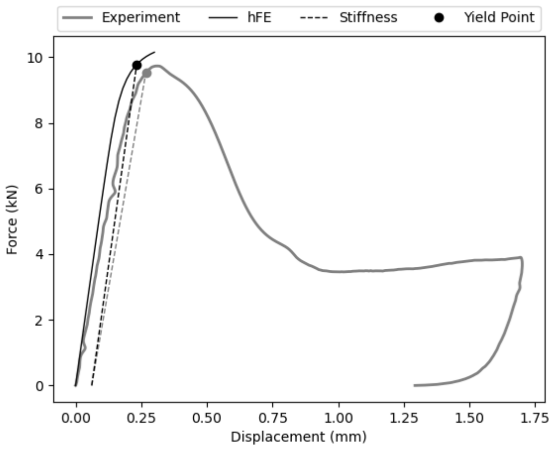

Figure 26: Sample 432

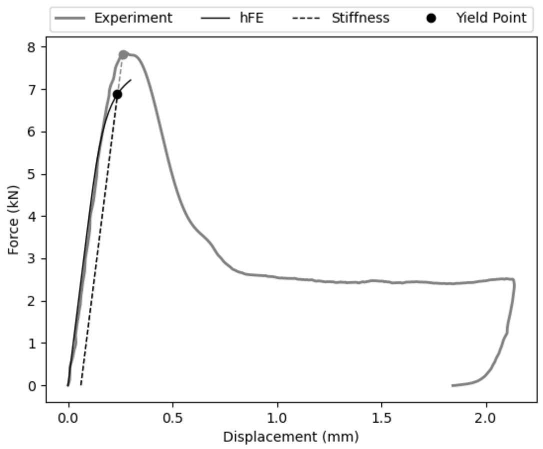

Figure 27: Sample 433

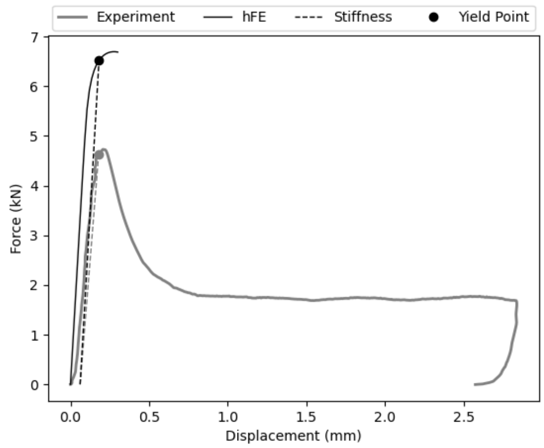

Figure 28: Sample 432

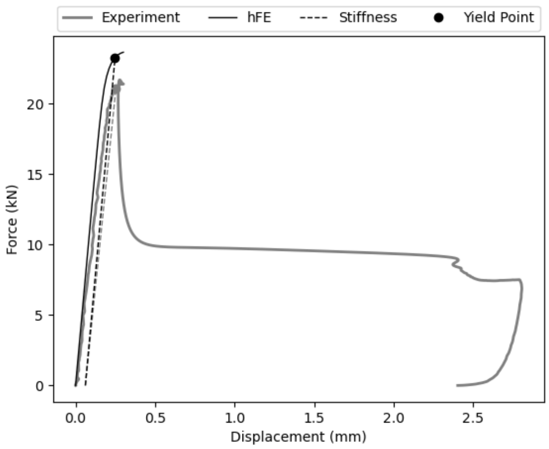

Figure 29: Sample 433

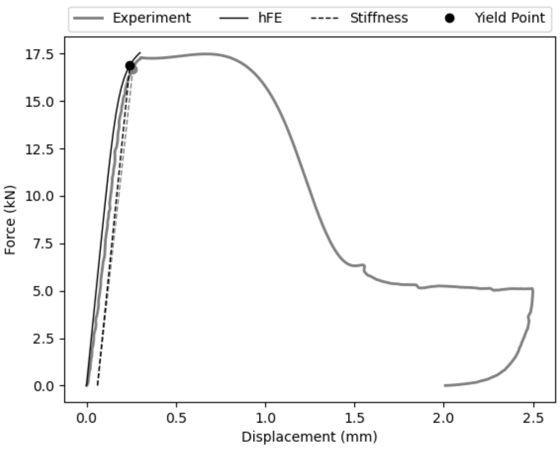

Figure 30: Sample 432

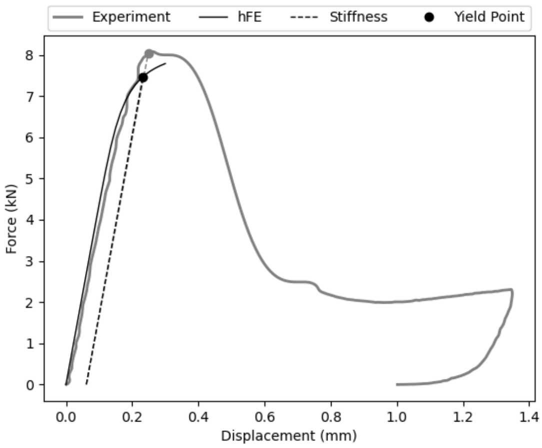

Figure 31: Sample 433

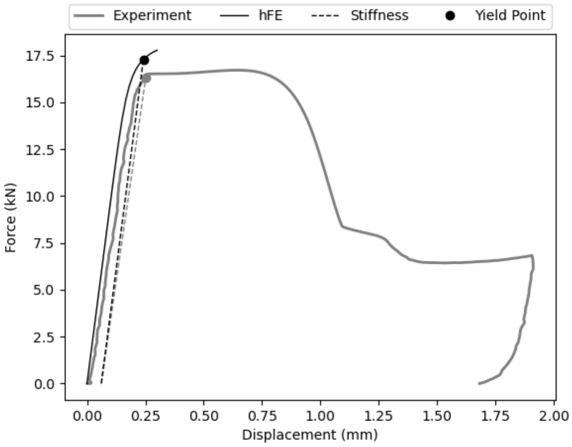

Figure 32: Sample 432

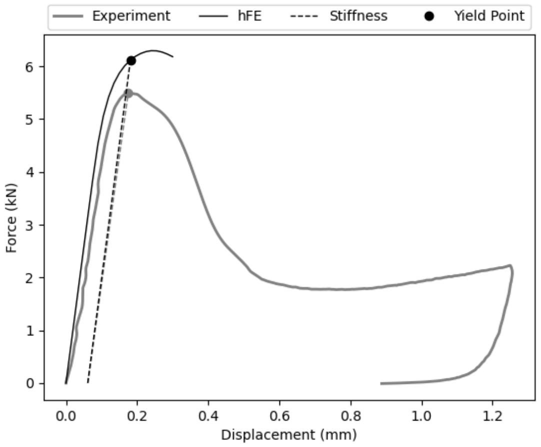

Figure 33: Sample 433

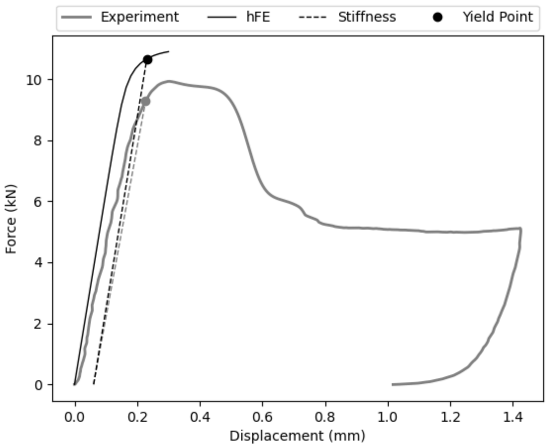

Figure 34: Sample 432

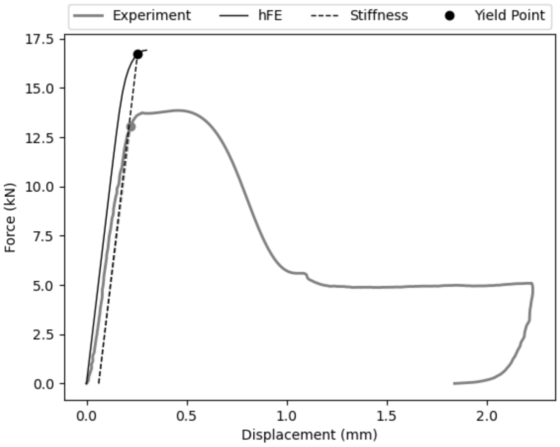

Figure 35: Sample 433

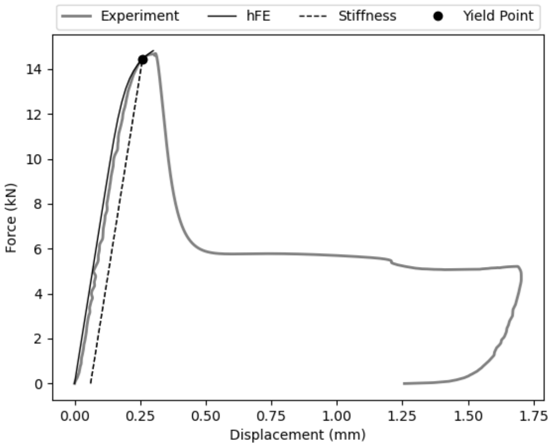

Figure 36: Sample 432

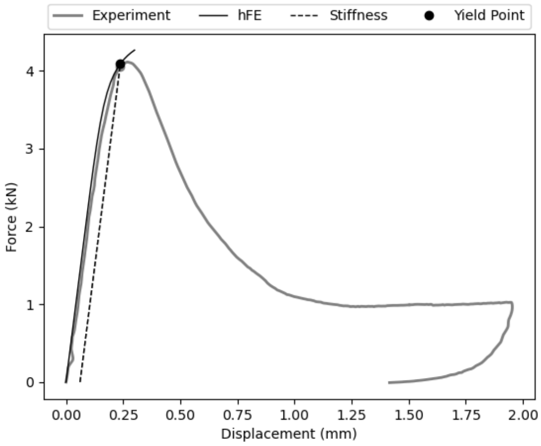

Figure 37: Sample 433

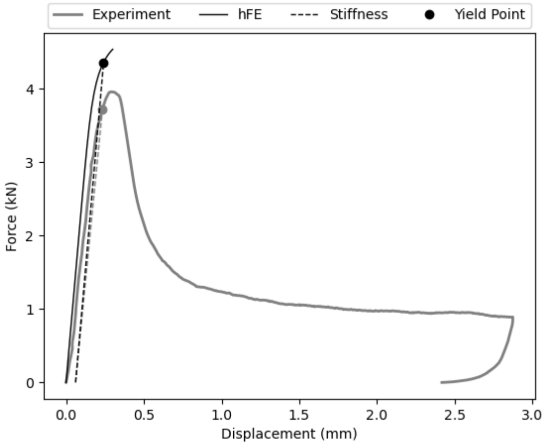

Figure 38: Sample 432

D. Registration and hFE Comparison

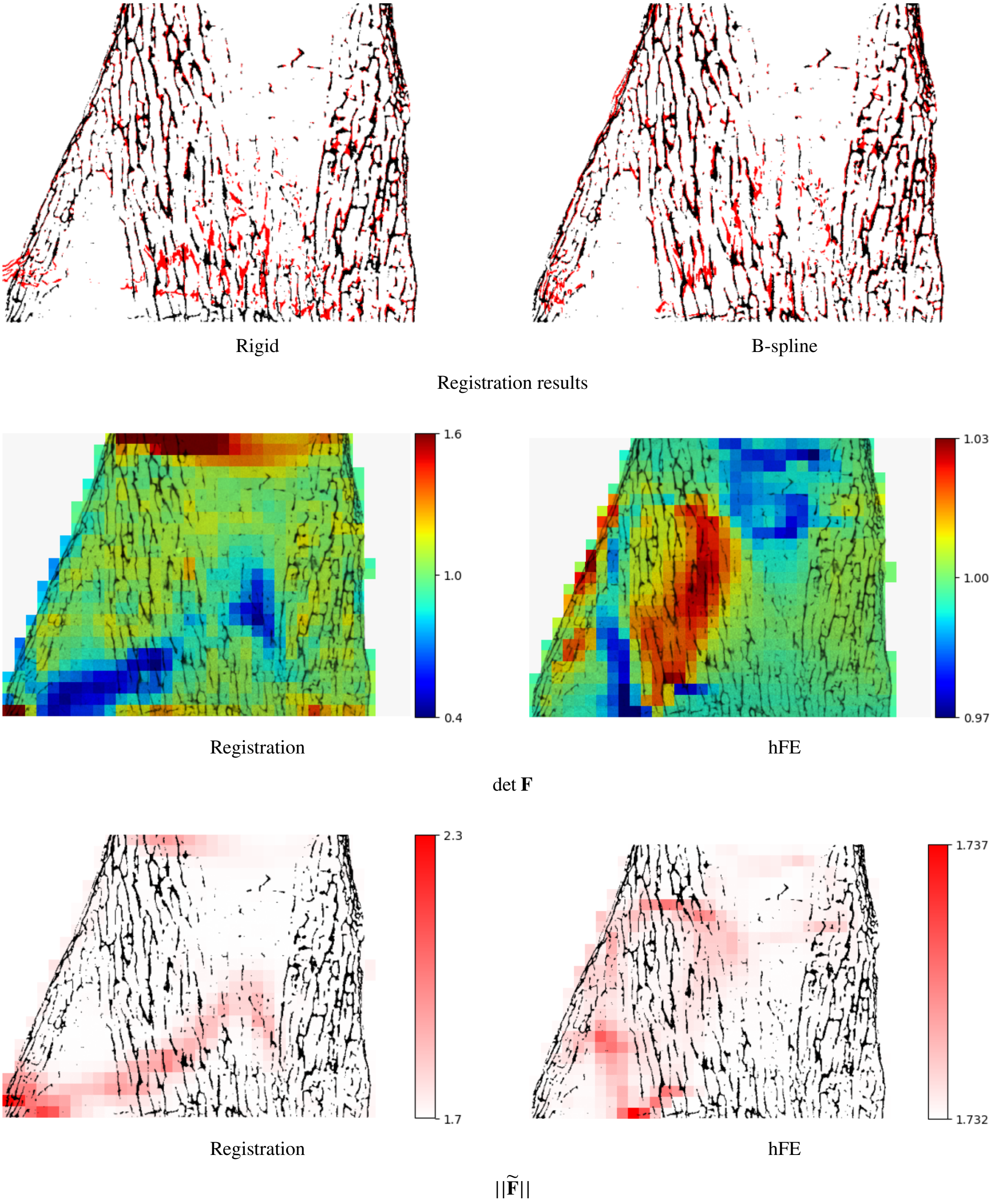

Figure 39: Sample 432

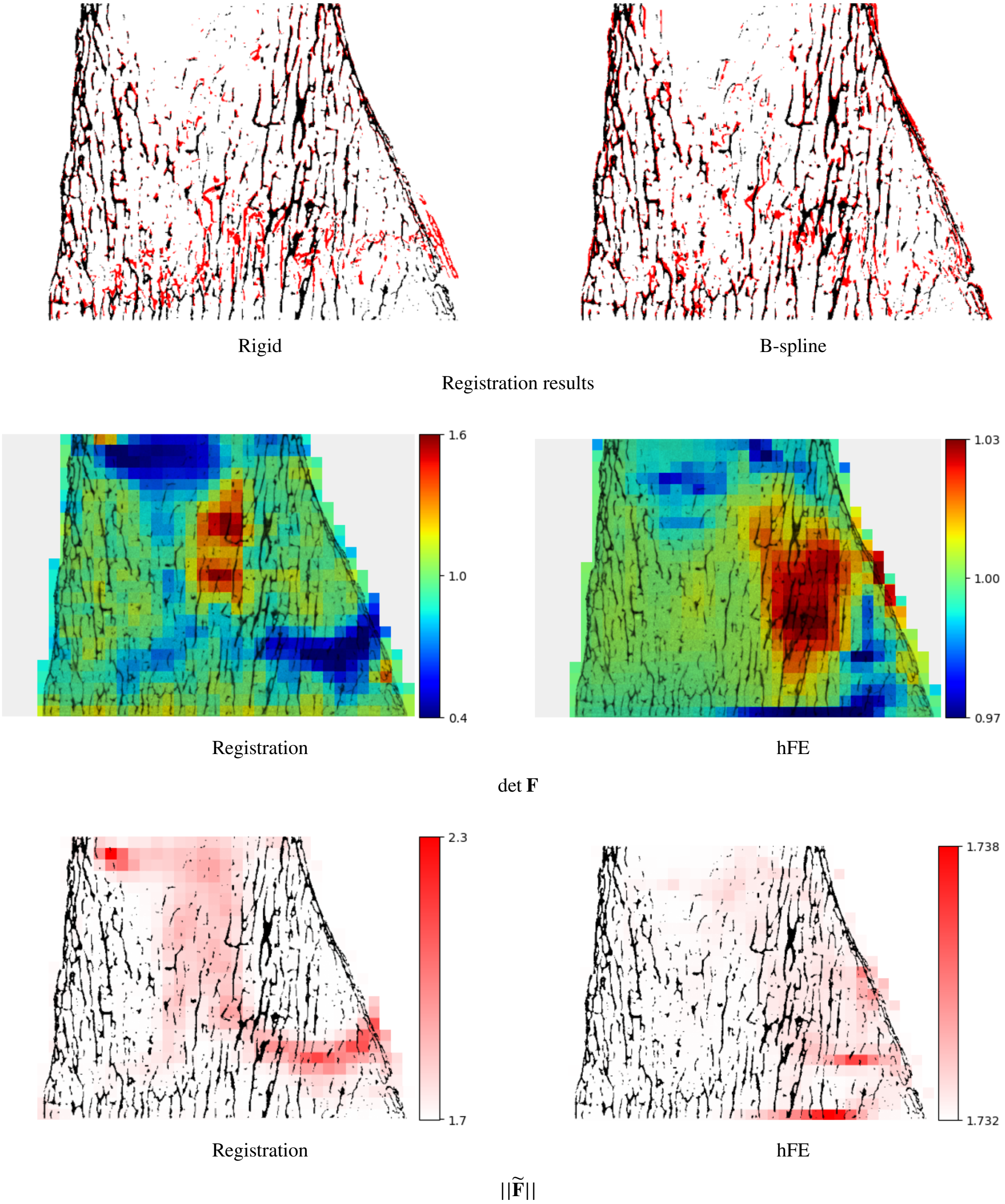

Figure 40: Sample 433

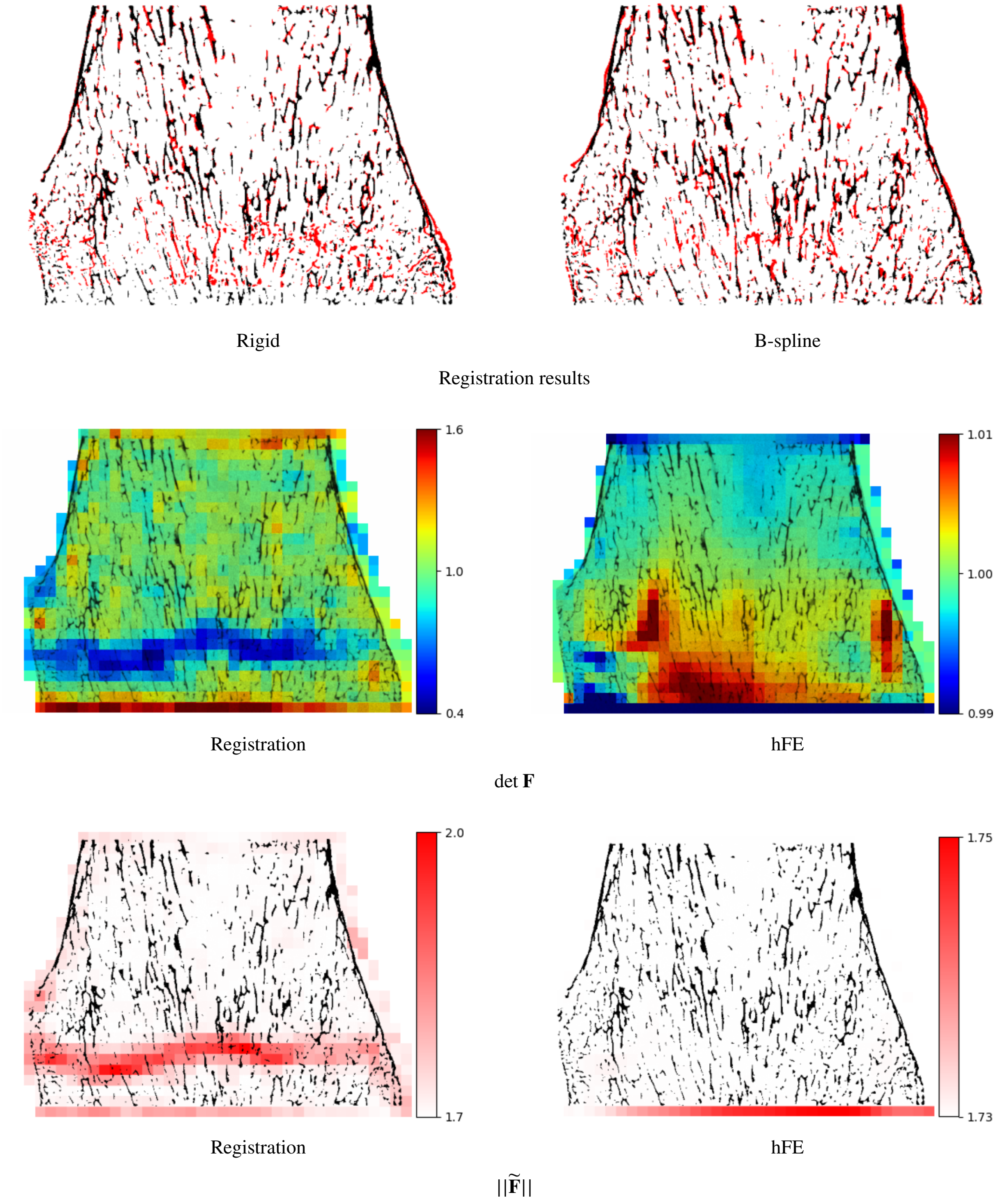

Figure 41: Sample 434

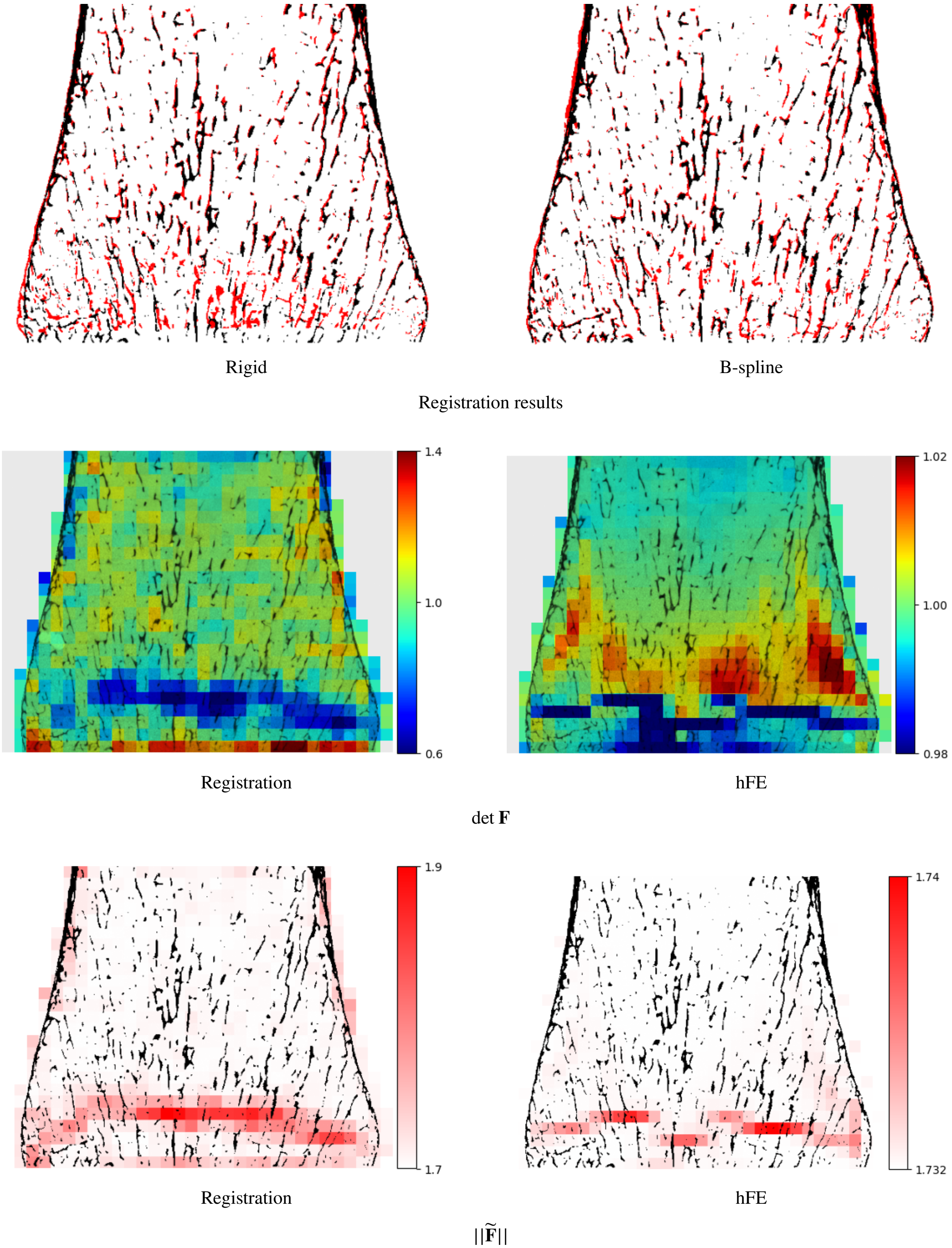

Figure 42: Sample 435

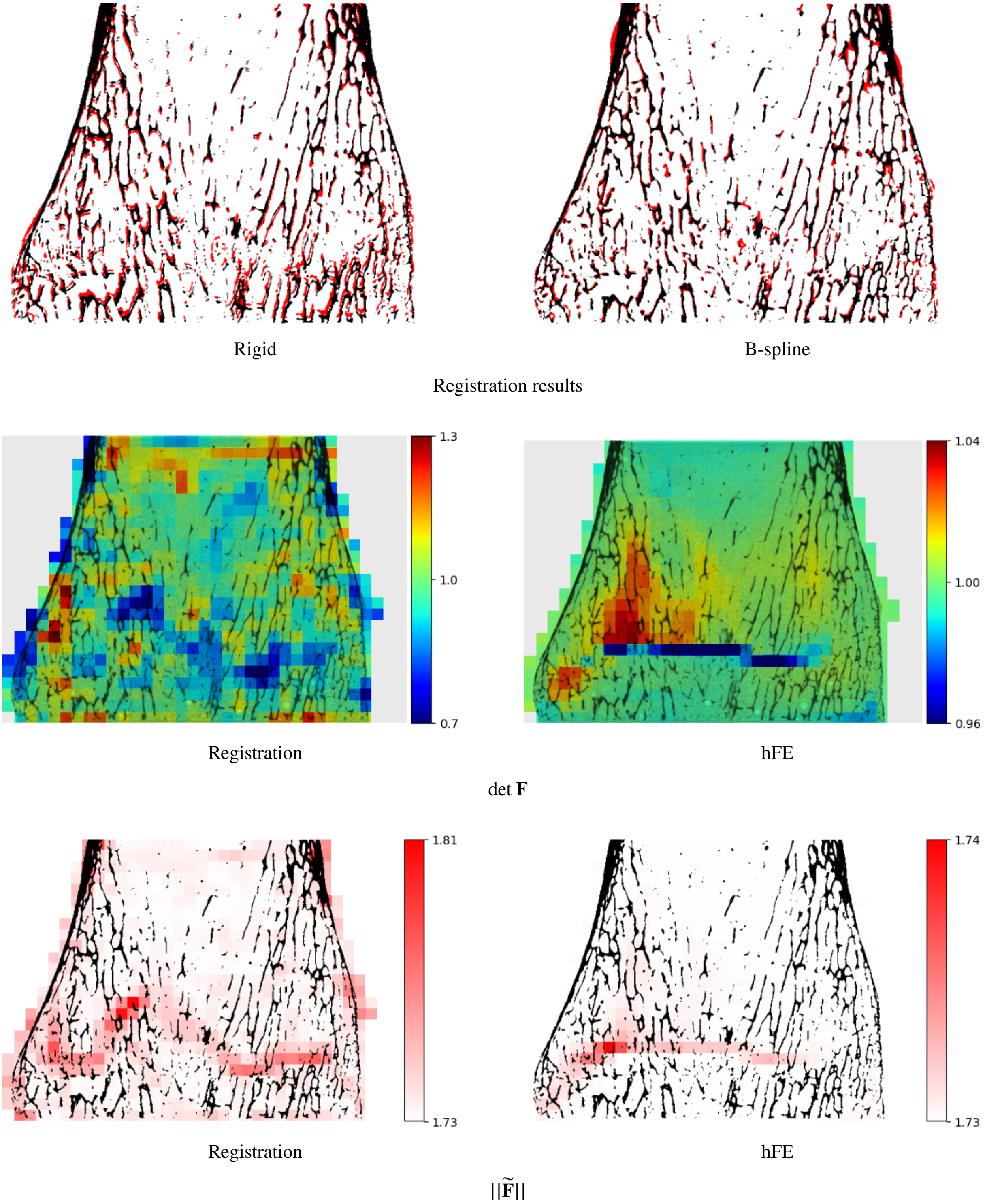

Figure 43: Sample 436

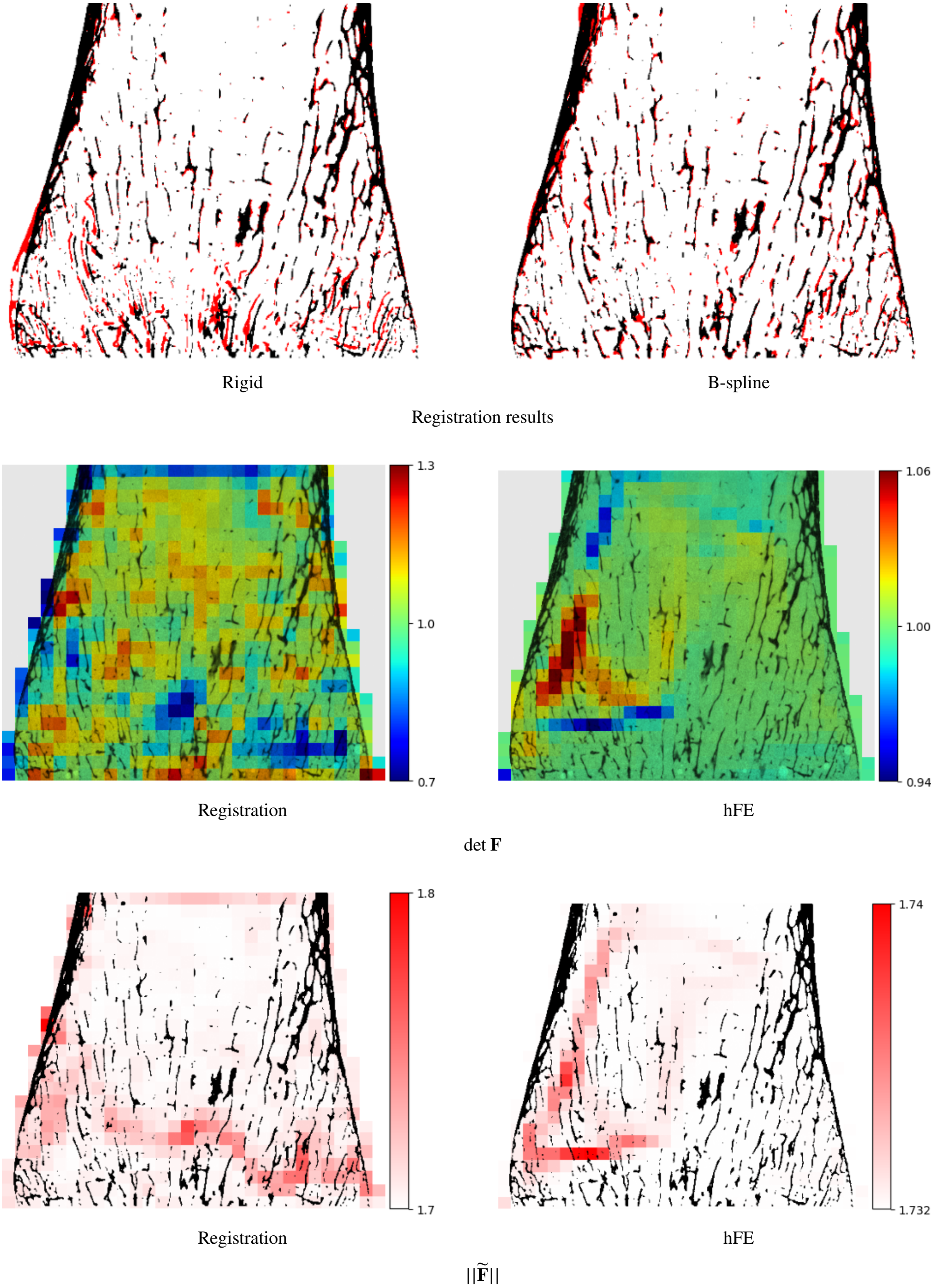

Figure 44: Sample 437

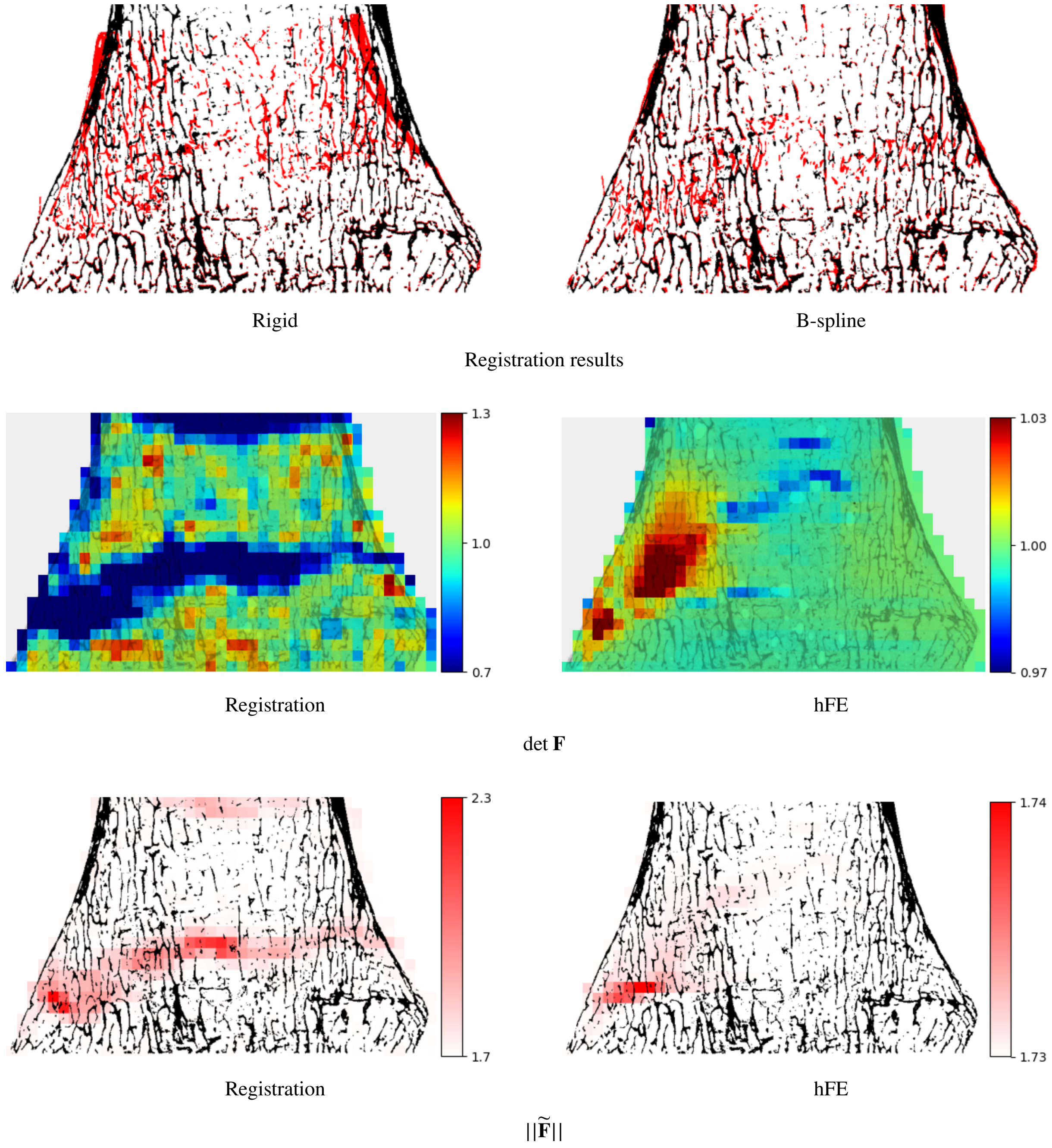

Figure 45: Sample 438

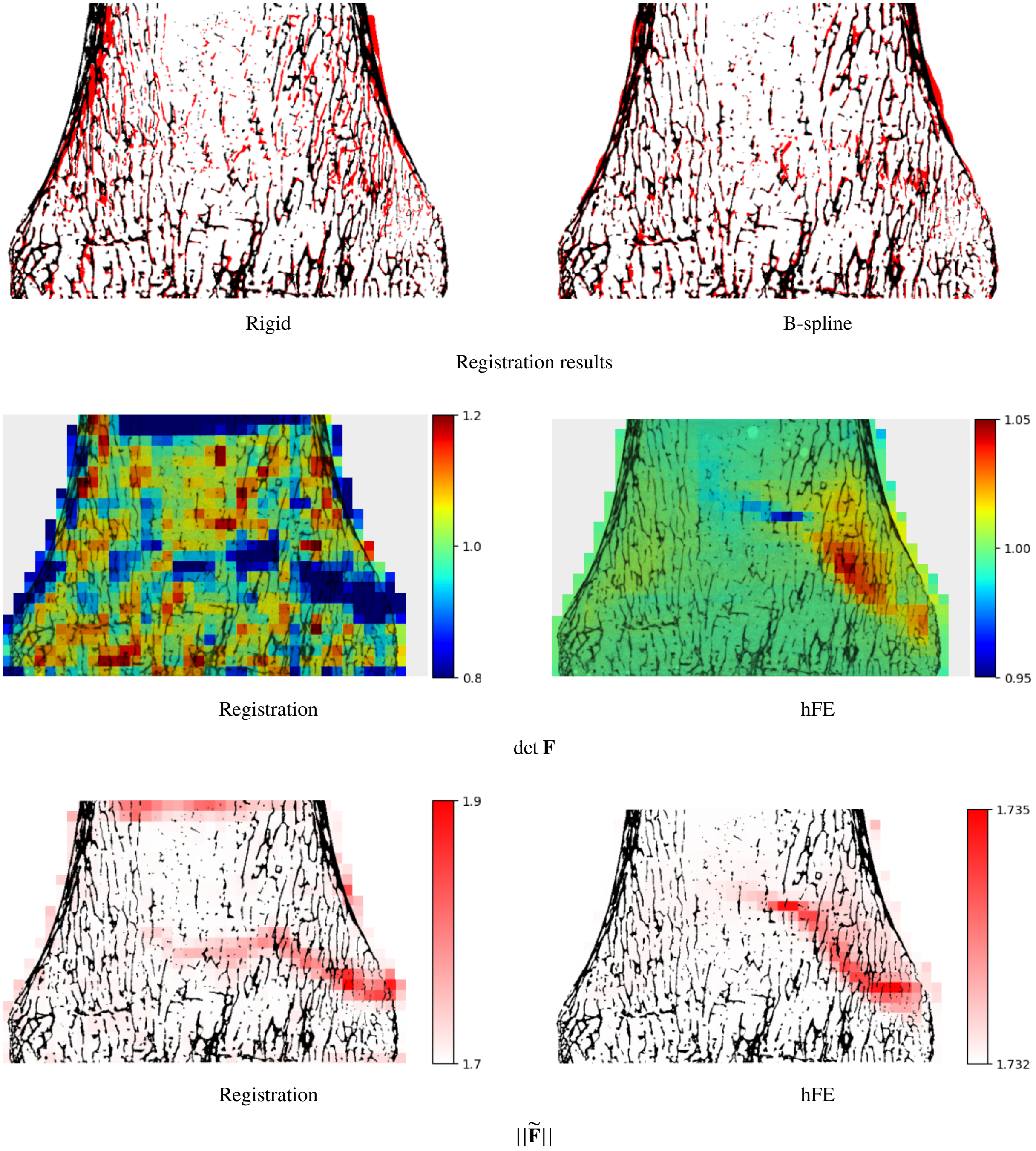

Figure 46: Sample 439

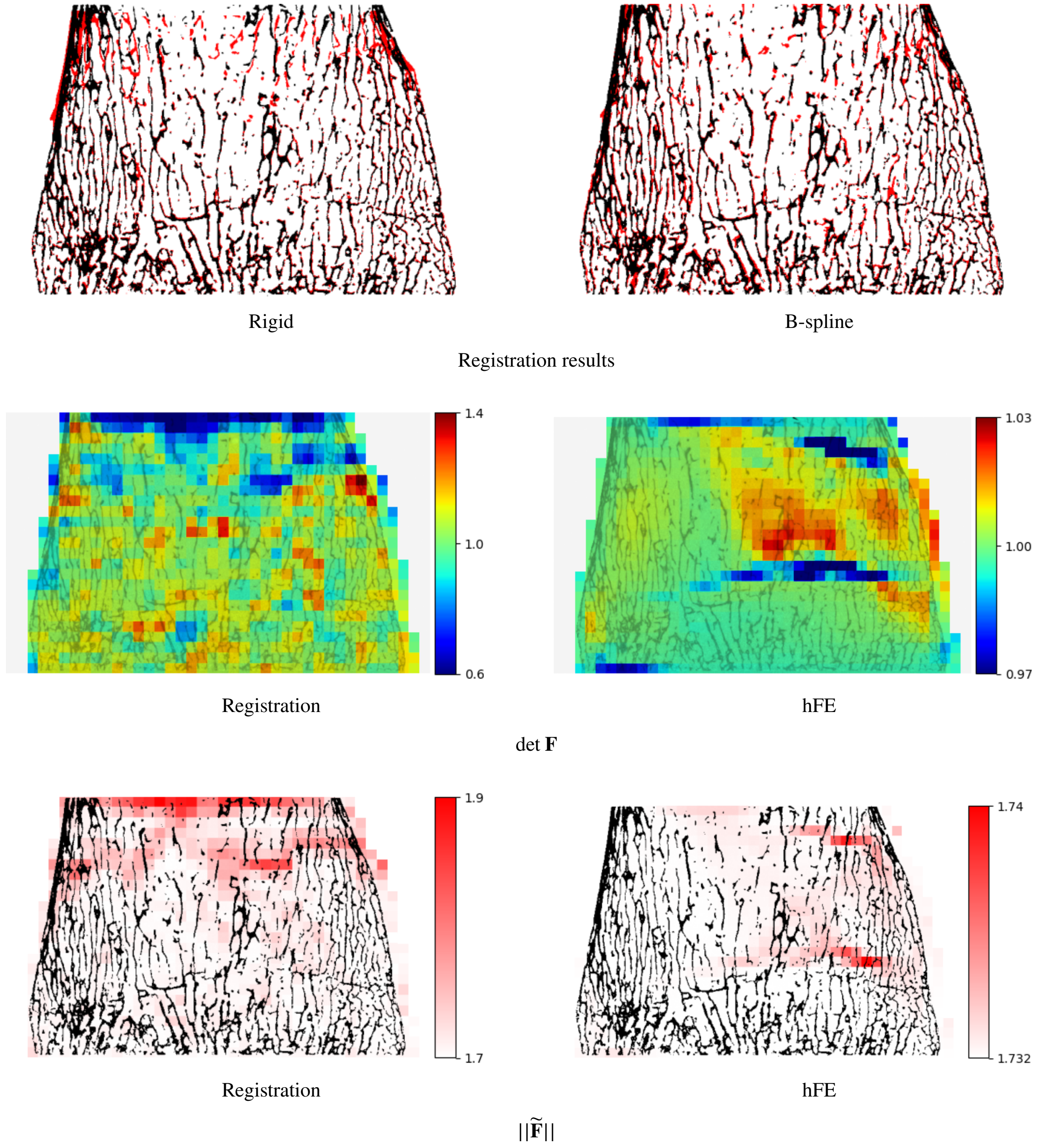

Figure 47: Sample 440

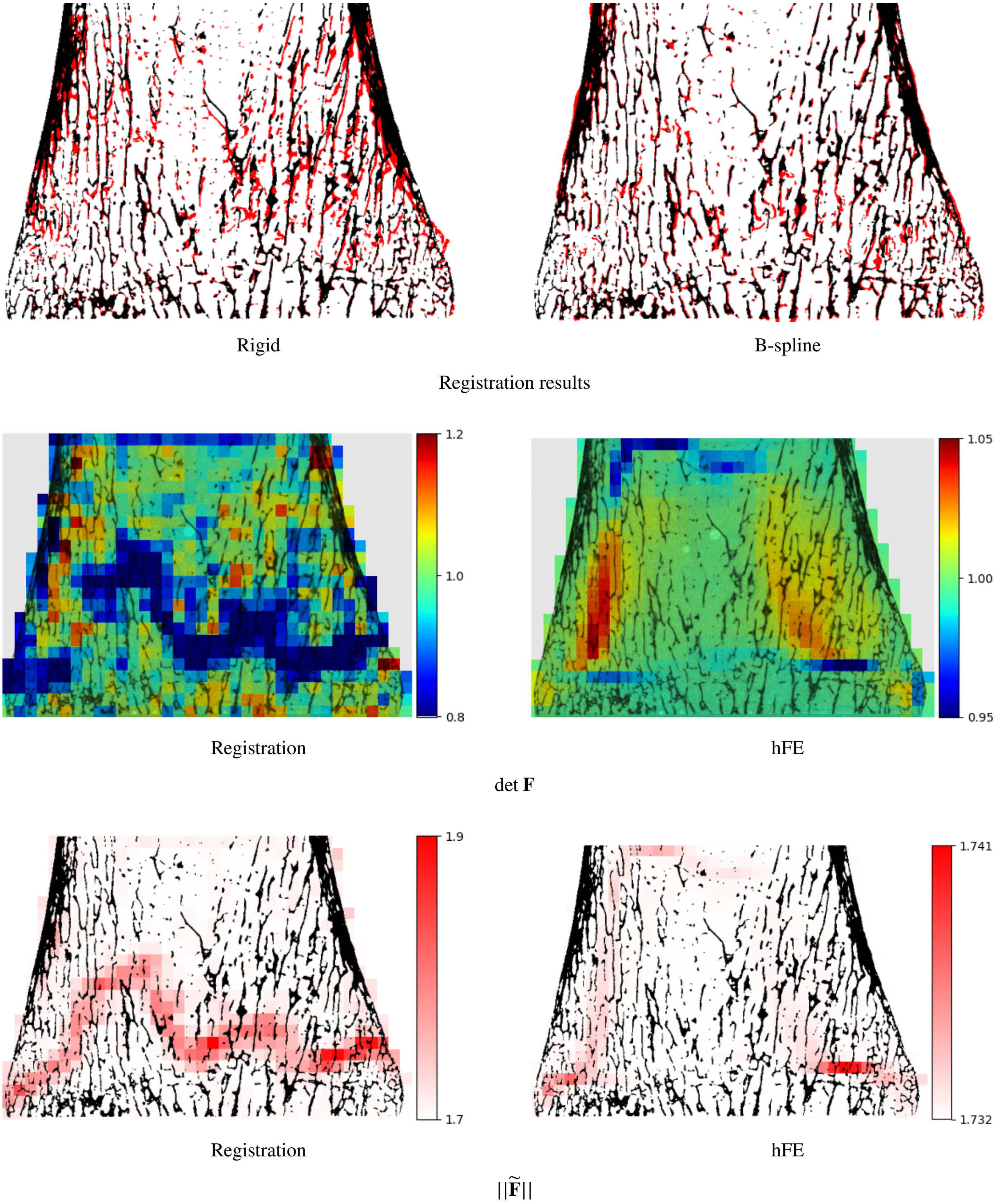

Figure 48: Sample 441

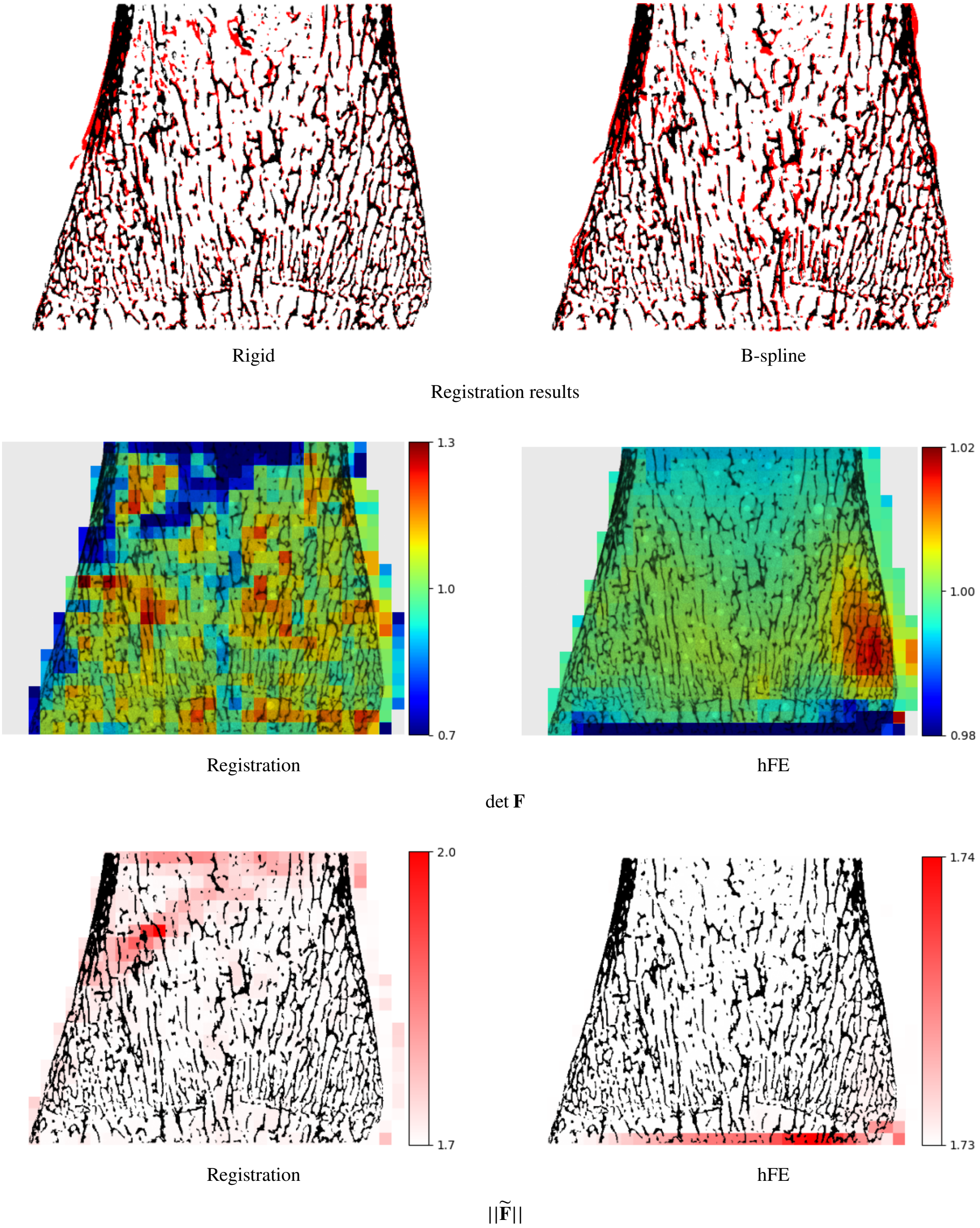

Figure 49: Sample 442

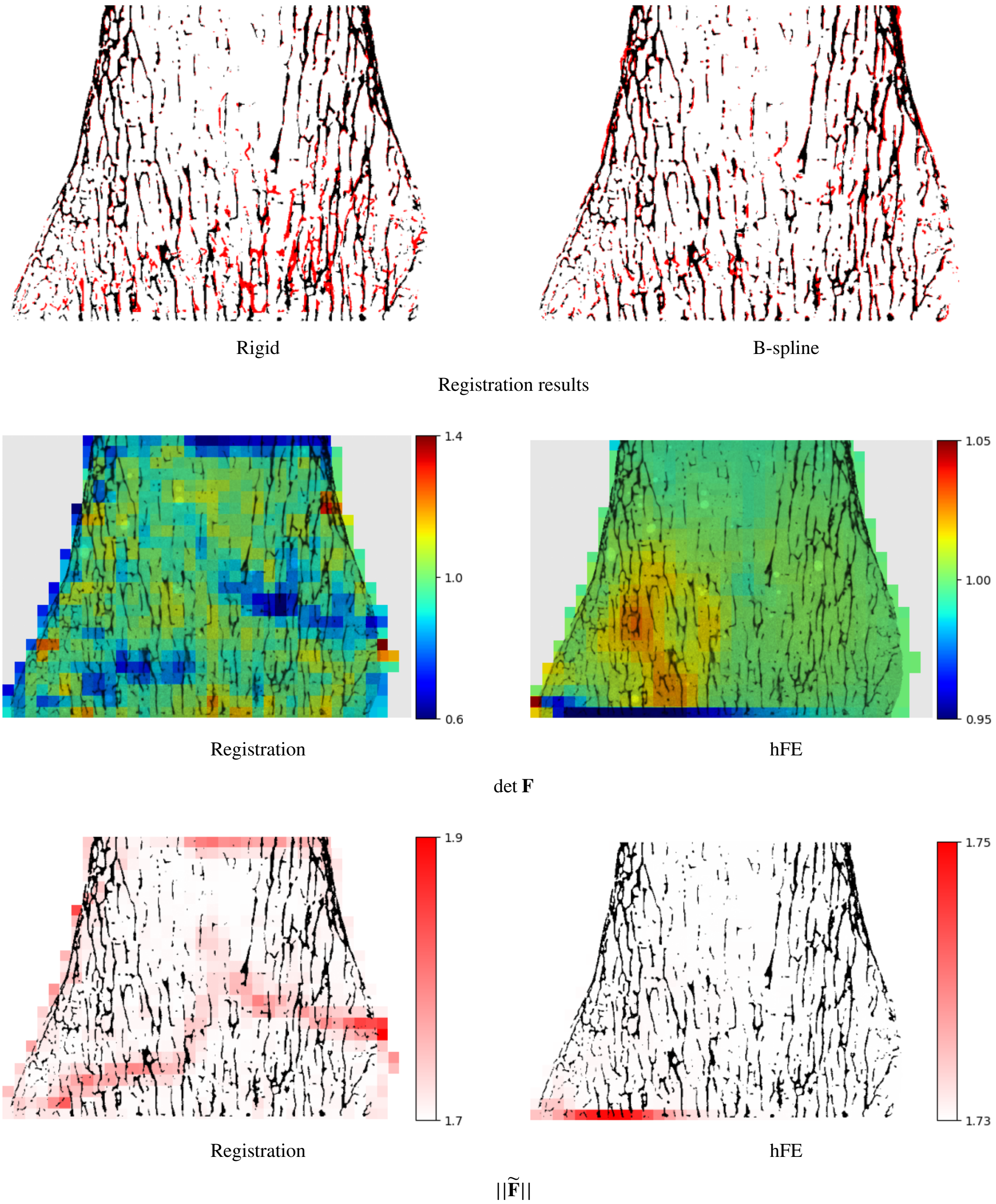

Figure 50: Sample 443

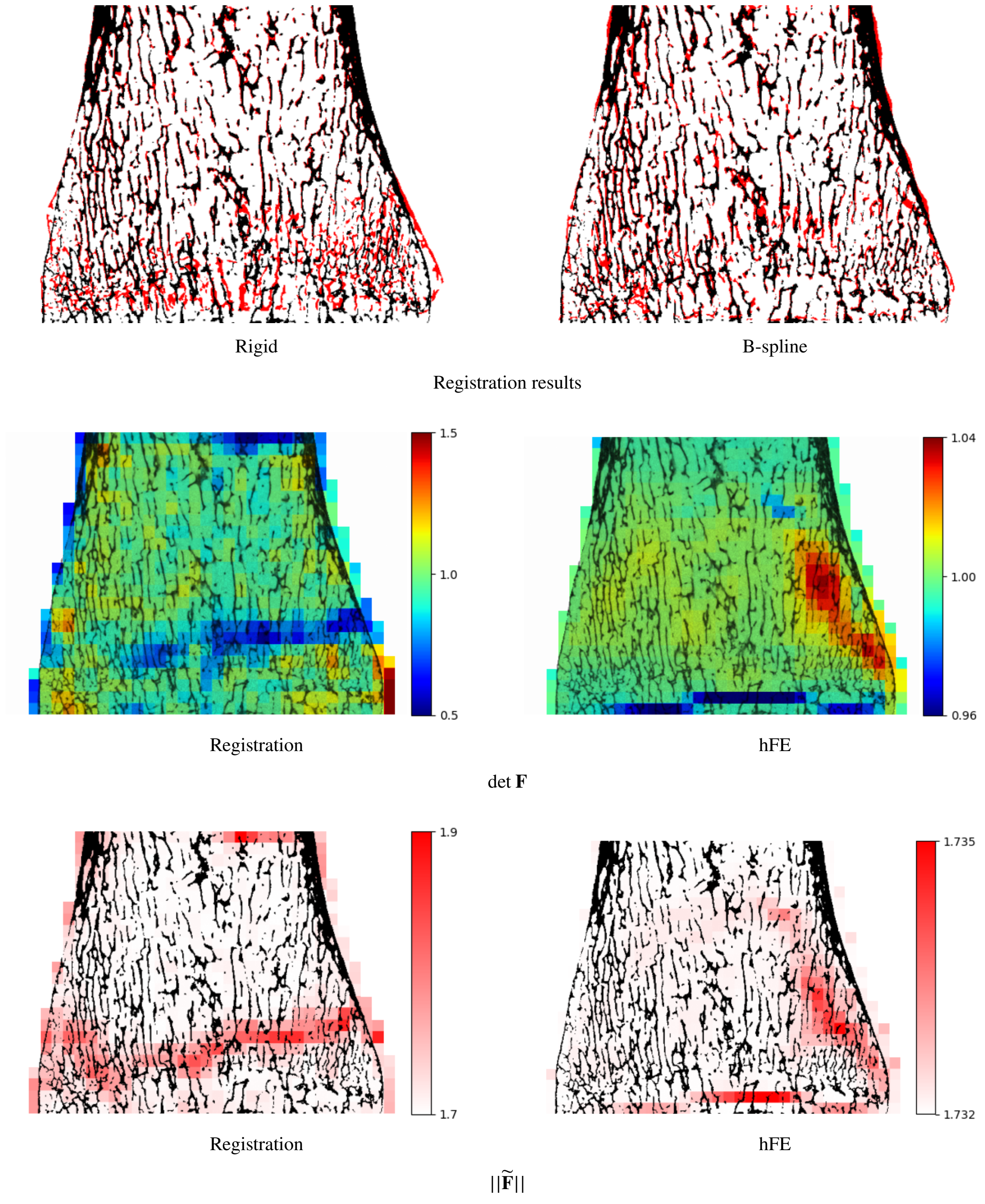

Figure 51: Sample 444

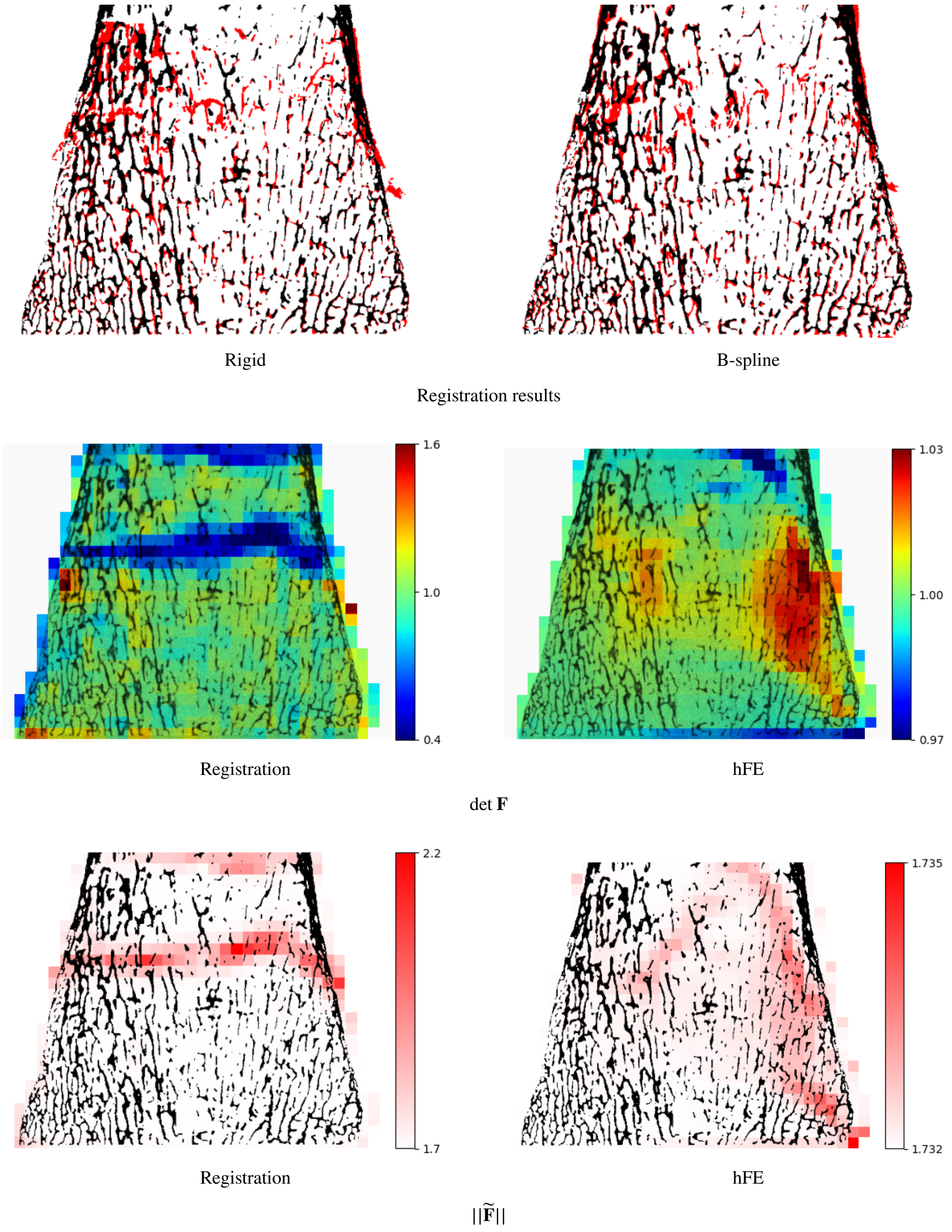

Figure 52: Sample 445

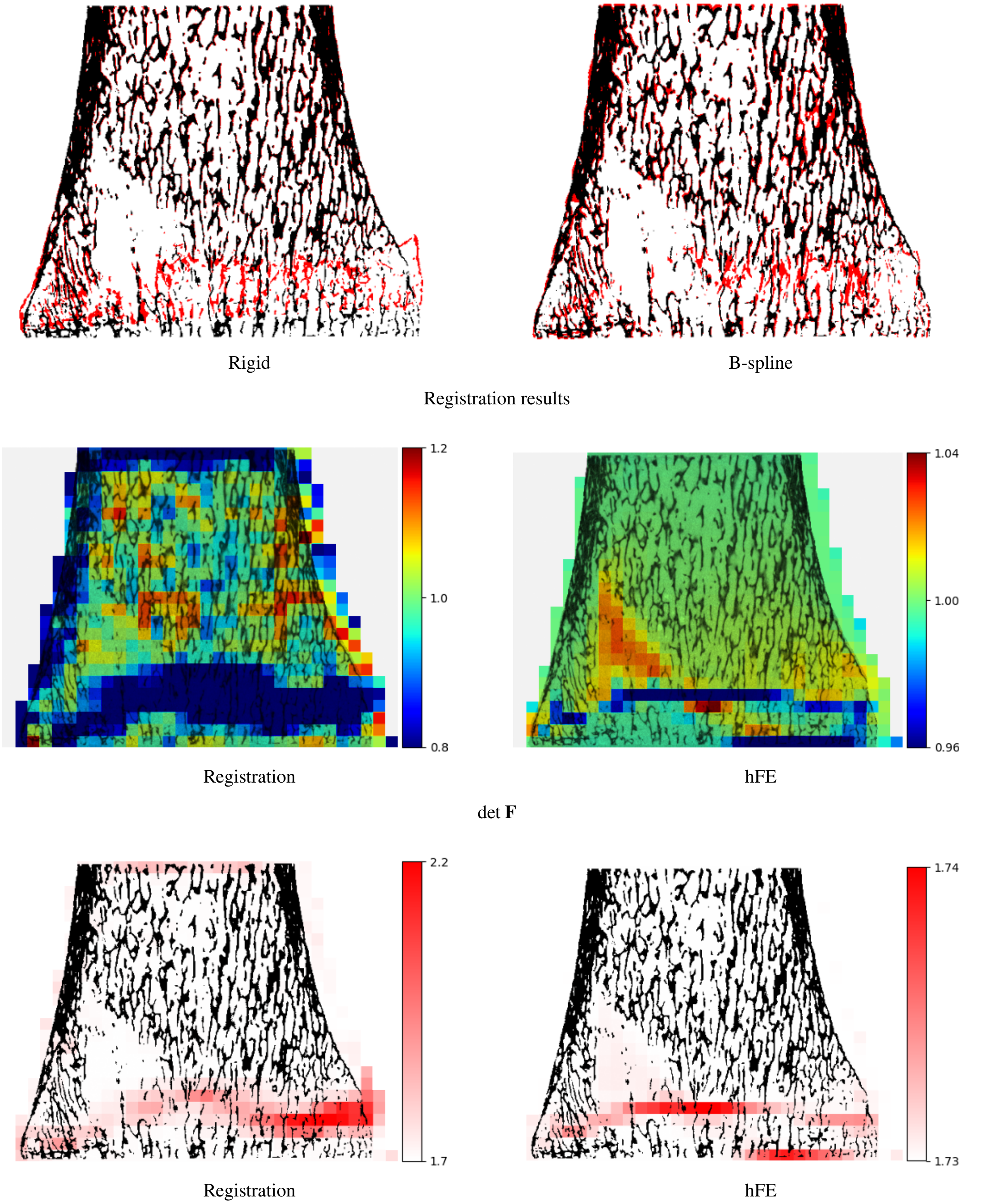

$||\widetilde{\mathbf{F}}||$

Figure 53: Sample 446

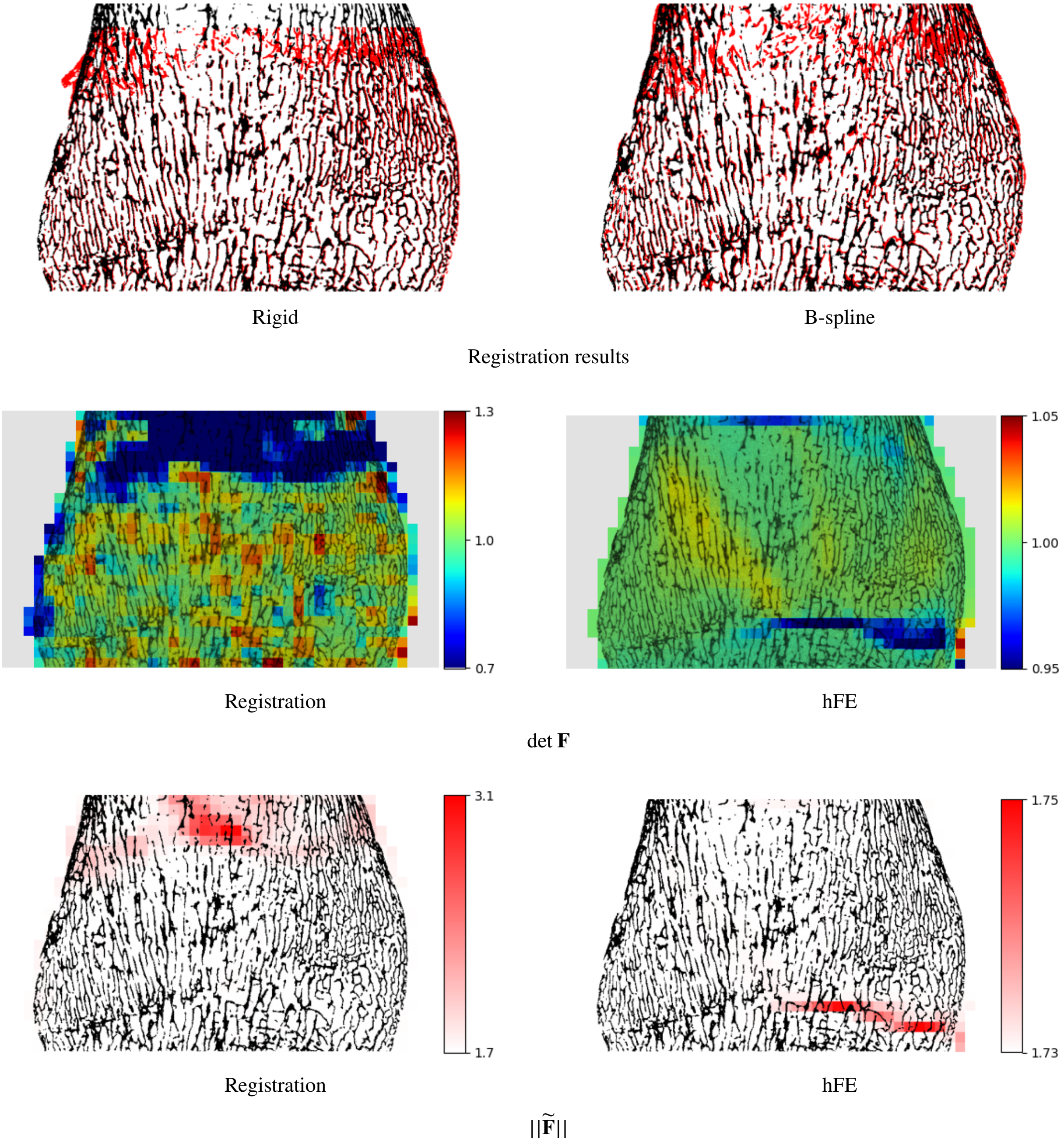

Figure 54: Sample 447

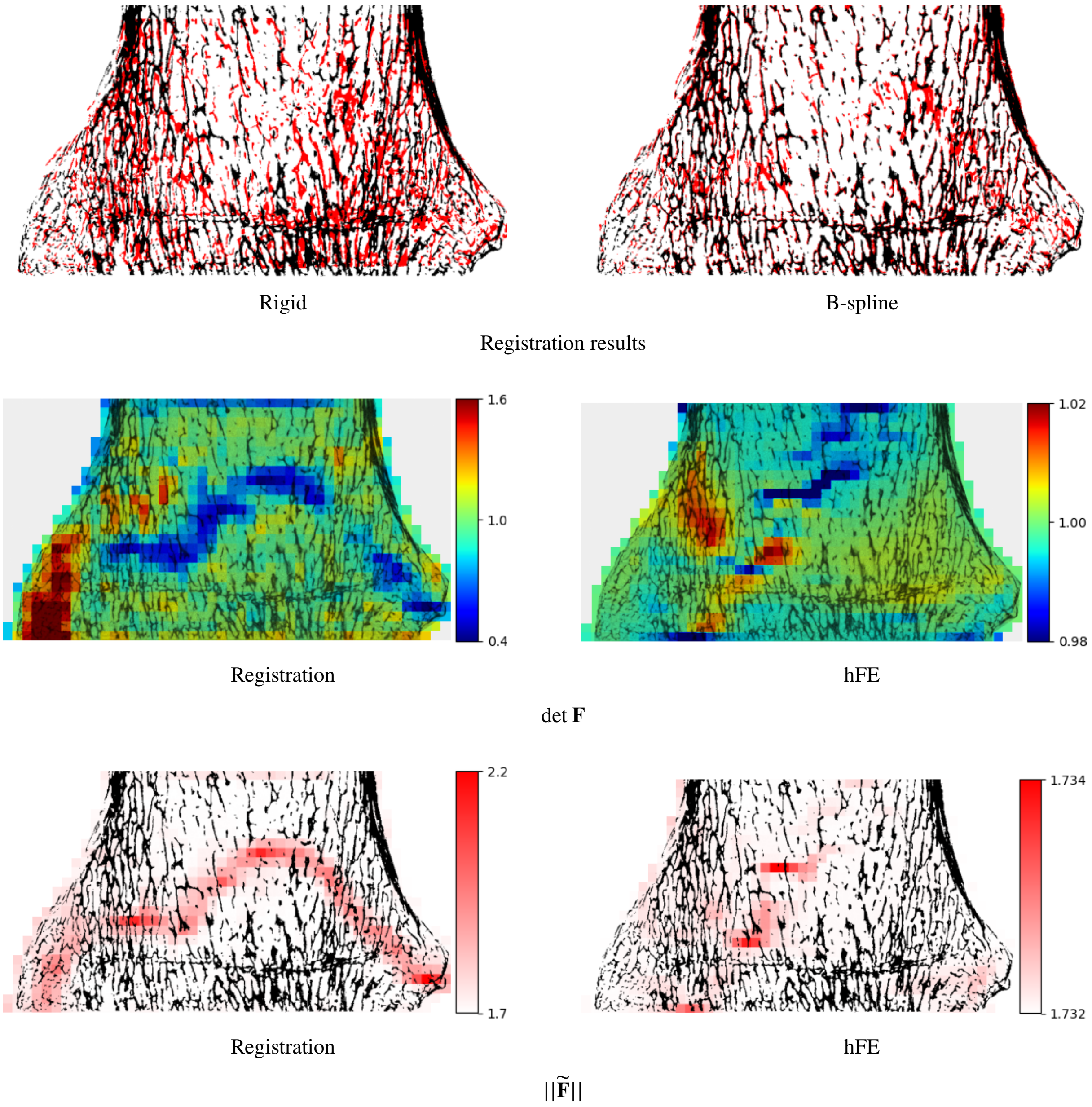

Figure 55: Sample 448

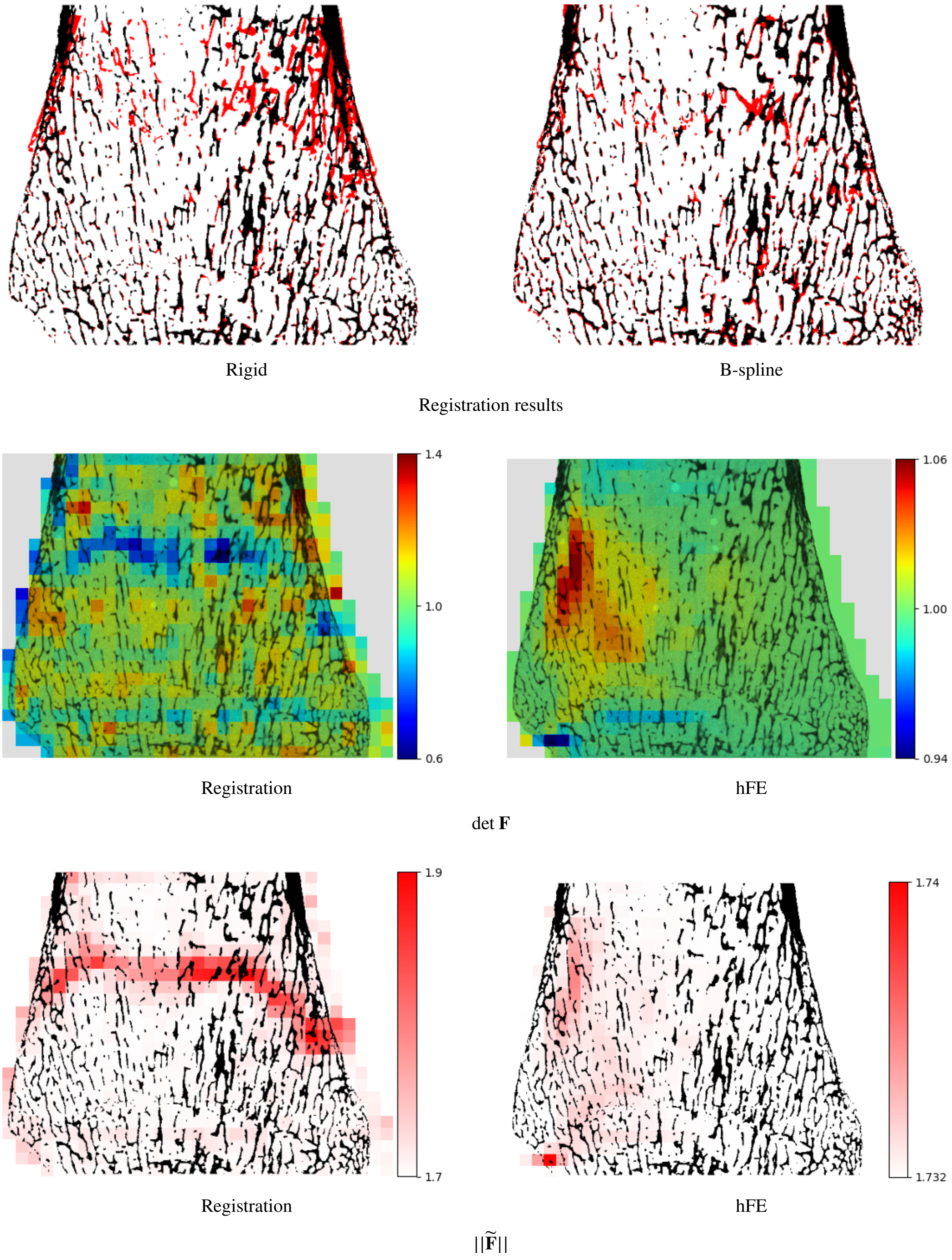

Figure 56: Sample 449

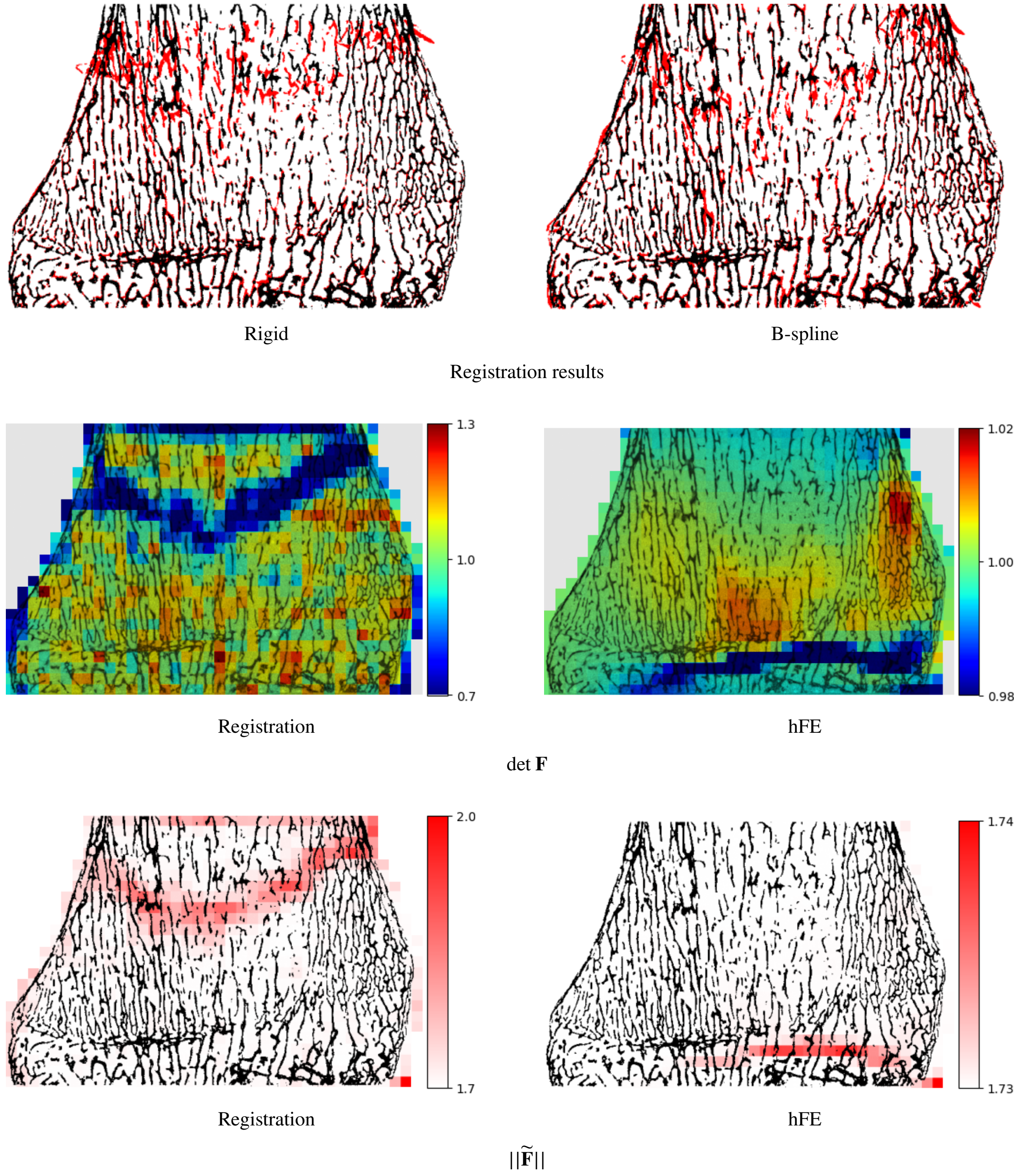

Figure 57: Sample 450

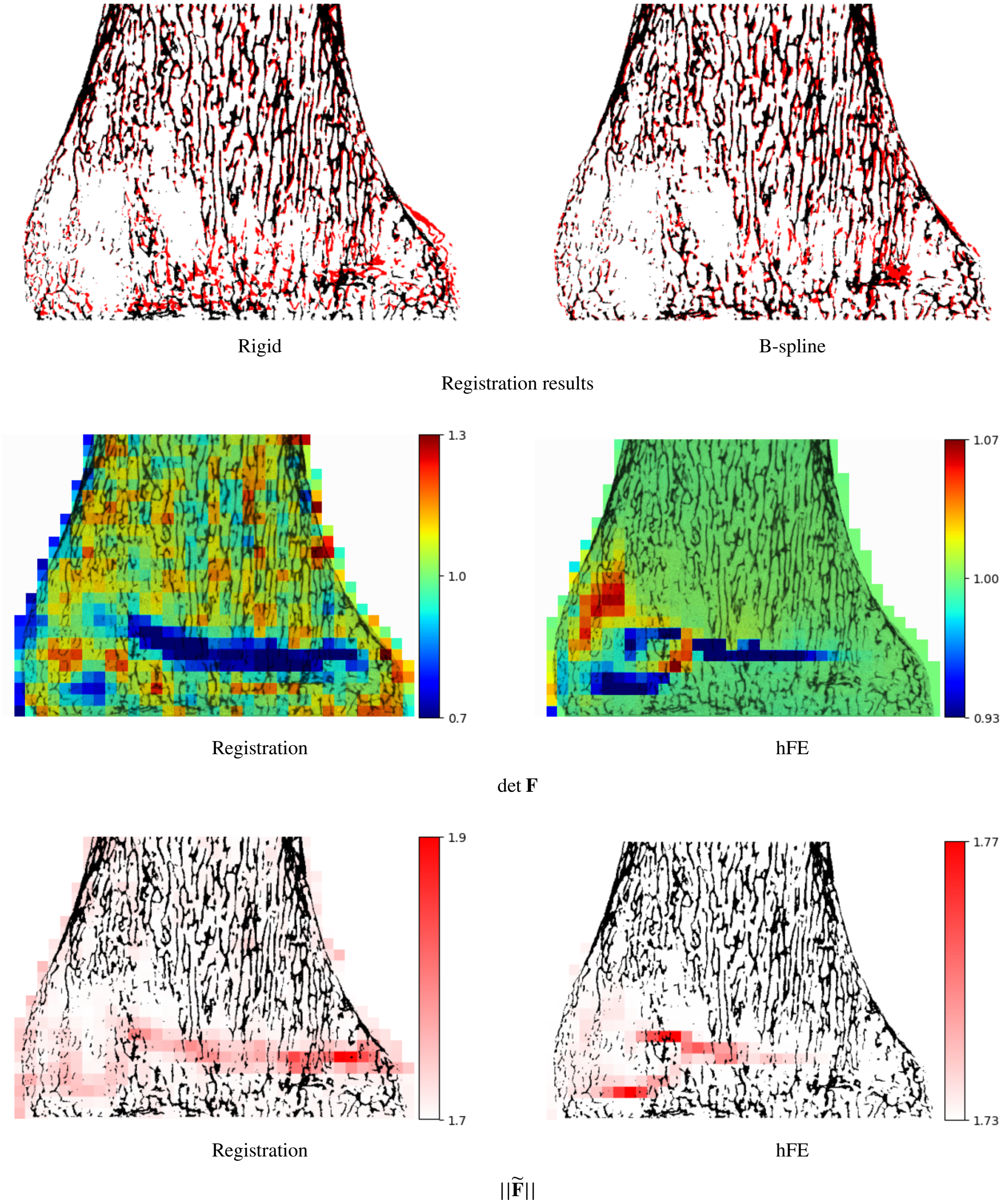

Figure 58: Sample 451

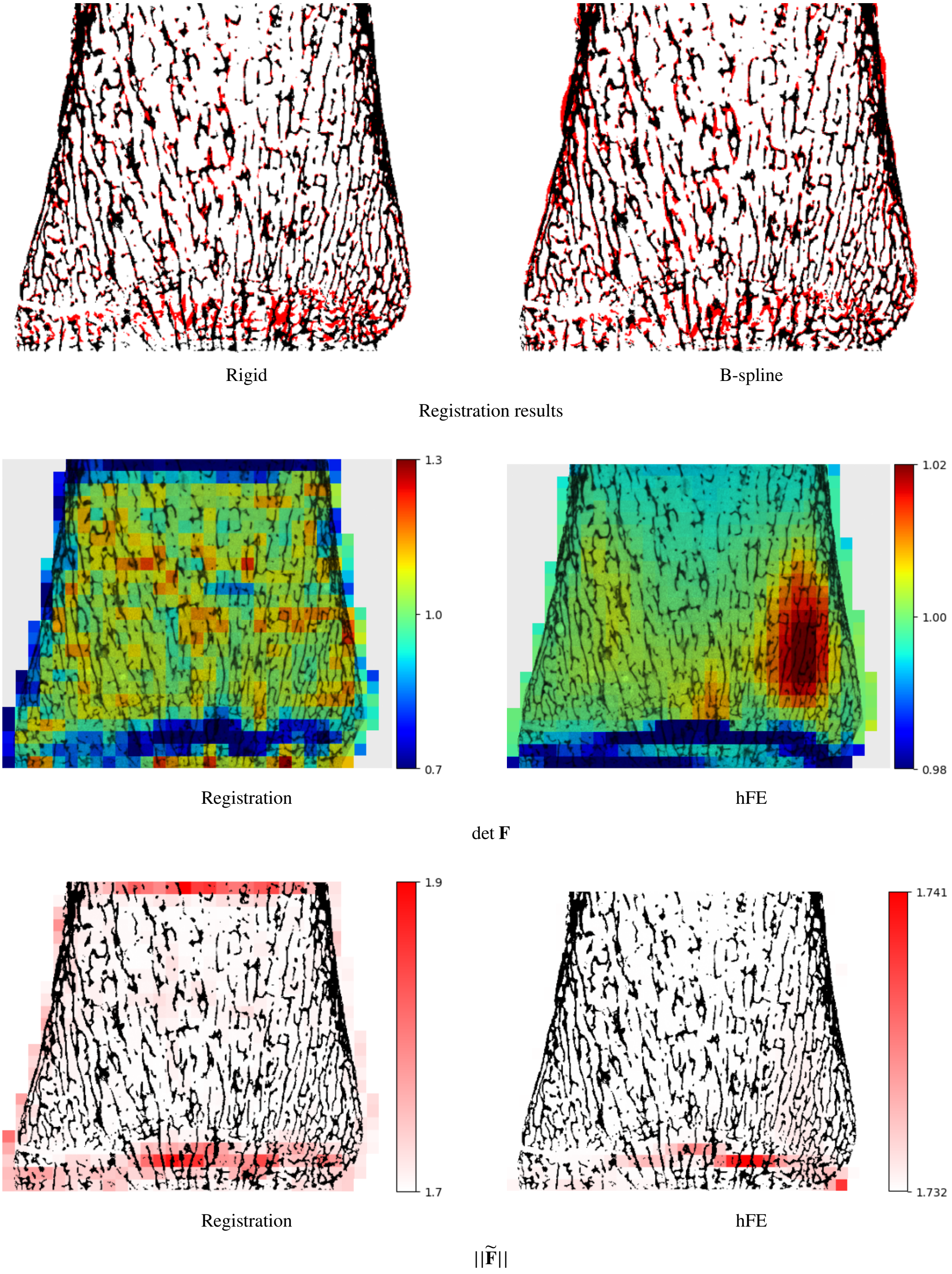

Figure 59: Sample 452

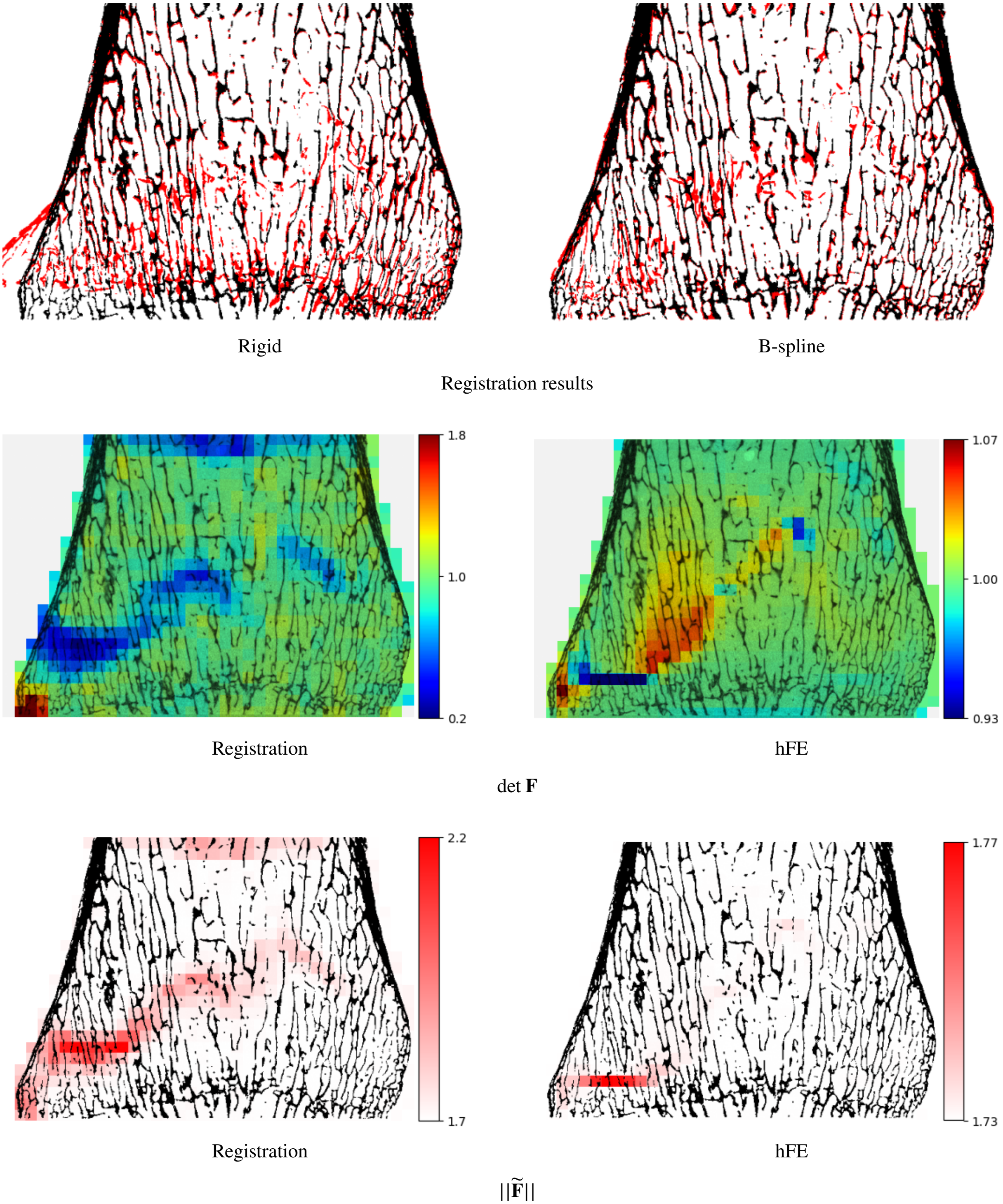

Figure 60: Sample 453

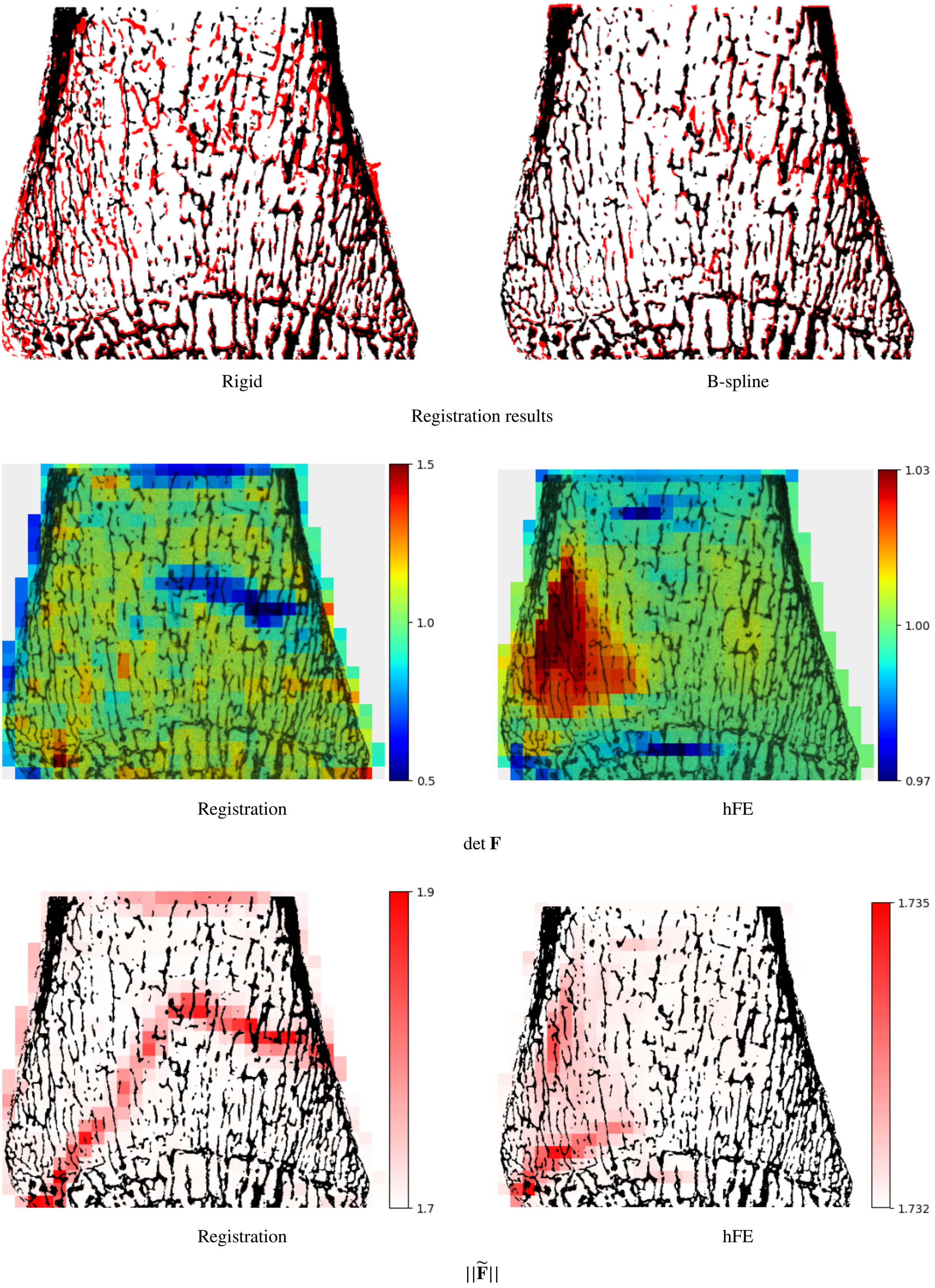

Figure 61: Sample 454

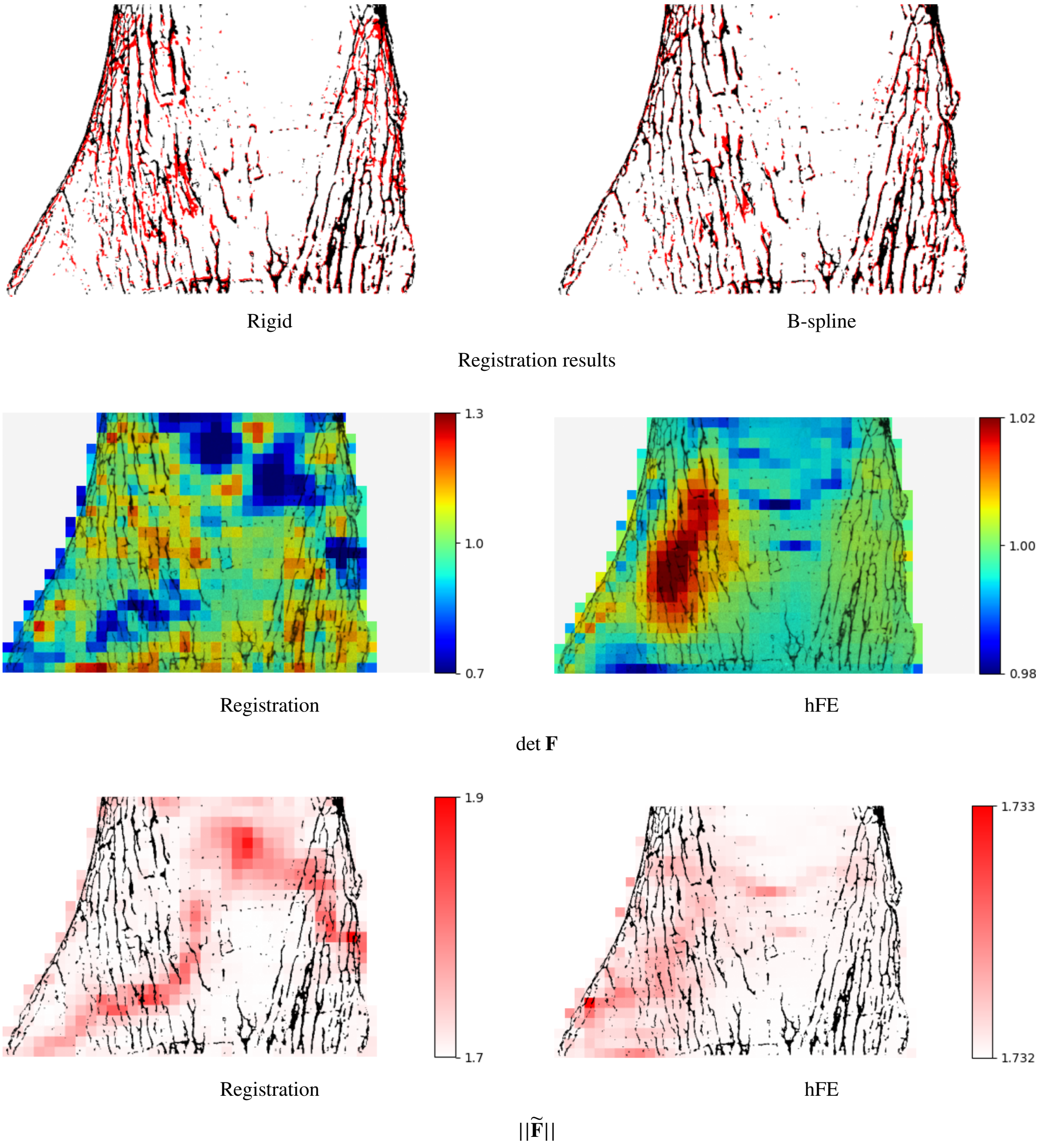

Figure 62: Sample 455

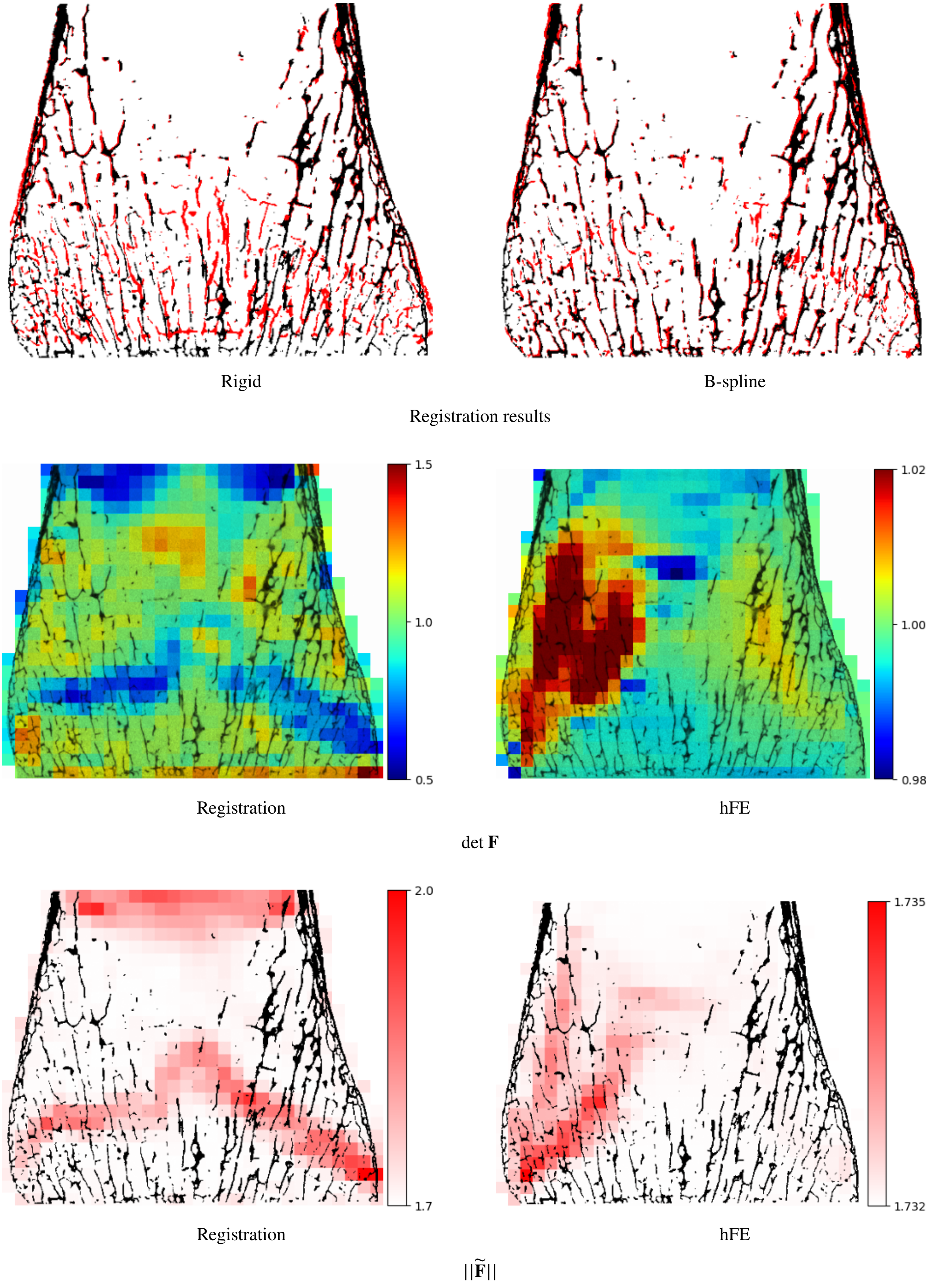

Figure 63: Sample 456
